# A comparative study of RNA-Seq and microarray data analysis on the two examples of rectal-cancer patients and Burkitt Lymphoma cells

**DOI:** 10.1371/journal.pone.0197162

**Published:** 2018-05-16

**Authors:** Alexander Wolff, Michaela Bayerlová, Jochen Gaedcke, Dieter Kube, Tim Beißbarth

**Affiliations:** 1 Dept. of Medical Statistics, University Medical Center Göttingen, Göttingen, Germany; 2 Dept. of General-, Visceral- and Pediatric Surgery, University Medical Center Göttingen, Göttingen, Germany; 3 Dept. of Hematology and Oncology, University Medical Center Göttingen, Göttingen, Germany; Luxembourg Institute of Health, LUXEMBOURG

## Abstract

**Background:**

Pipeline comparisons for gene expression data are highly valuable for applied real data analyses, as they enable the selection of suitable analysis strategies for the dataset at hand. Such pipelines for RNA-Seq data should include mapping of reads, counting and differential gene expression analysis or preprocessing, normalization and differential gene expression in case of microarray analysis, in order to give a global insight into pipeline performances.

**Methods:**

Four commonly used RNA-Seq pipelines (STAR/HTSeq-Count/edgeR, STAR/RSEM/edgeR, Sailfish/edgeR, TopHat2/Cufflinks/CuffDiff)) were investigated on multiple levels (alignment and counting) and cross-compared with the microarray counterpart on the level of gene expression and gene ontology enrichment. For these comparisons we generated two matched microarray and RNA-Seq datasets: Burkitt Lymphoma cell line data and rectal cancer patient data.

**Results:**

The overall mapping rate of STAR was 98.98% for the cell line dataset and 98.49% for the patient dataset. Tophat’s overall mapping rate was 97.02% and 96.73%, respectively, while Sailfish had only an overall mapping rate of 84.81% and 54.44%. The correlation of gene expression in microarray and RNA-Seq data was moderately worse for the patient dataset (ρ = 0.67–0.69) than for the cell line dataset (ρ = 0.87–0.88). An exception were the correlation results of Cufflinks, which were substantially lower (ρ = 0.21–0.29 and 0.34–0.53). For both datasets we identified very low numbers of differentially expressed genes using the microarray platform. For RNA-Seq we checked the agreement of differentially expressed genes identified in the different pipelines and of GO-term enrichment results.

**Conclusion:**

In conclusion the combination of STAR aligner with HTSeq-Count followed by STAR aligner with RSEM and Sailfish generated differentially expressed genes best suited for the dataset at hand and in agreement with most of the other transcriptomics pipelines.

## Introduction

Transcriptomics as an area in the research field of functional genomics has always been a key player for identifying interactions and regulations of gene expression. Over the last two decades it was common practice to use microarrays for any investigation in transcriptomics. Within the last ten years the next generation sequencing (NGS) and especially RNA sequencing (RNA-Seq), became widely available [1]. These technologies are gradually replacing microarrays, when analyzing and identifying complex mechanism in gene expression. Decreasing running costs, higher dynamic range of expression and higher accuracy in low abundance measurements [2] are the main factors for this fast development of NGS and increasing use of RNA-Seq over microarray.

The versatility in using RNA-Seq, like discovering novel small RNAs (smRNA), microRNA (miRNA), long-non-coding RNAs (lncRNA) or alternative splicing events [3], is a further factor for an increasing popularity of this profiling approach. Another advantage is the currently highly discussed variant calling [4] [5] [6] based on RNA-Seq data, which makes this technology even more attractive. The developments of new technologies, like Pacific Bioscience or Nanopore [7], can further contribute in the field of RNA-Seq and transcriptomics in form of more detailed annotation databases in the future.

A typical application for RNA-Seq is the differential gene expression analysis. First, millions of short reads are produced, which are mapped to a reference genome. Subsequently, the amount of reads mapping to a genomic feature of interest (for example a gene, transcript or exon) is measured as the abundance of these features [8]. The abundance per feature is used as an input for differential expression analysis.

Still, microarrays are widely used because of their lower costs compared to the RNA-Seq technology. Moreover, there are large and well maintained repositories, such as ArrayExpress [9] and Gene Expression Omnibus (GEO) [10], that have collected the microarray data over long time periods. RNA-Seq data collections are increasing in GEO and the The Cancer Genome Atlas (TCGA, https://cancergenome.nih.gov/).

While the preprocessing and analysis steps of microarray data are mostly standardized, the establishment of RNA-Seq data analysis methodology and standards is still ongoing in the field of transcriptomics. A lot of efforts have been performed into method comparison studies to change this [11] [12] [13] [14]. The quality evaluation of different RNA-Seq (pre-)processing methods are one important step to establish a quality standard. Great effort in this field have been accomplished, for instance by Sequencing Quality Control (SEQC) consortium [11] and has already been done for microarrays years ago in the MAQC-I and MAQC-II projects [15] [16].

We aim to investigate commonly used RNA-Seq pipelines on multiple levels (alignment, counting) and cross-compare the results with the microarray counterpart on the level of gene expression and gene ontology enrichment. For these evaluations we generated two matched microarray and RNA-Seq datasets: rectal cancer (RC) patient data (good versus bad prognosis patients) and Burkitt Lymphoma (BL2) cell line data (control versus stimulated cells).

## Materials and methods

### Burkitt Lymphoma cell-line data (BL2)

BL2 cells were cultivated as described previously at cell densities between 2 × 10^5^ and 1 × 10^6^ cells/ml [17]. For stimulation studies, cells were cultured in cell culture medium supplemented with 10 mM HEPES at 1 × 10^6^ cells/ml and incubated with B-cell activating factor (BAFF) for up to 24 hrs instead of 9hrs [18]. RNA was isolated with RNAeasy Plus Mini Kit (Qiagen) according to the manufacturer’s instructions and labeled using Affymetrix GeneChip IVT Labelling Kit (Affymetrix). Fragmentation and hybridization on Human ST1.0 Arrays were processed according to manufacturer’s recommendations by the TAL (UMG, Germany). Microarray based profiling was performed using Affymetrix GeneChip Human Gene 1.0 ST array in three independent replicates of the experiment with the stimulated versus unstimulated cell line. For RNA-Seq, single-end sequencing on an Illumina HiSeq 2000 machine with the poly-A capturing protocol with 43 base pairs read length was used. The RNA was isolated using Trizol reagent including a DNase I (Roche, Mannheim, Germany) digestion step and Library preparation was performed using the TruSeq Stranded Sample Preparation Kit (Illumina, RS-122-2201) starting from 1000 ng of total RNA. Accurate quantitation of cDNA libraries was performed using the QuantiFluor TM dsDNA System (Promega). The size range of nal cDNA libraries was determined applying the SS-NGS-Fragment 1–6000 bp Kit on the Fragment Analyzer from Advanced Analytical (320 bp). cDNA libraries were amplified and sequenced by using the cBot and the HiSeq2000 from Illumina. The BL2 dataset is accessible through GEO Series accession number GSE99768 for the RNA-Seq dataset and GSE100112 for the microarray data.

### Rectal cancer patient data (RC)

The rectal cancer patient dataset consists of 10 patients from a clinical study at the Surgery department of the University Medical Center Göttingen collected over a longer time. Patients were chosen based on the follow-up time and development of a distant metastasis. First a balanced sample size of five versus five patients with and without a metastatic event was intended. A later development of metastasis of one of the good prognosis patients changed the sample size to 6 versus 4 patients. The study is approved from the Ethic commission of the University medical centre Göttingen, ethic number: 9/8/08. Biopsies were immediately stored in RNAlater (Qiagen, Hilden, Germany). Subsequently, for microarray RNA was isolated using TRIzol (Invitrogen, Carlsbad, CA) according to the manufacturer´s instructions. Nucleic acid quantity, quality and purity were determined using a spectrophotometer (Nanodrop, Rockland, DE) and a 2100 Bioanalyzer (Agilent Technologies, Palo Alto, CA). 1 μg of total RNA was labeled with Cy3 using the Low RNA Input Fluorescent Linear Amplification Kit according to the manufacturer’s recommendations (Agilent Technologies, Santa Clara, CA). Quantity and efficiency of the labeled amplified cRNA were determined using the NanoDrop ND-1000 UV-VIS Spectrophotometer version 3.2.1. 1.5 mg of Cy3-labeled cDNA was hybridized to an oligonucleotide-based Whole Human Genome Microarray (4x44K, Agilent Technologies) and incubated at 65°C for 17 hours. Slides were washed and scanned using an Agilent G2565BA scanner.

For RNA-Seq single-end sequencing for 50 base pair reads the RNA was isolated using Trizol reagent including a DNase I (Roche, Mannheim, Germany) digestion step. Library preparation for RNA-Seq was performed using the TruSeq Stranded Sample Preparation Kit (Illumina, RS-122-2201) starting from 1000 ng of total RNA. Accurate quantitation of cDNA libraries was performed using the QuantiFluor TM dsDNA System (Promega). The size range of nal cDNA libraries was determined applying the SS-NGS-Fragment 1–6000 bp Kit on the Fragment Analyzer from Advanced Analytical (320 bp). cDNA libraries were amplified and sequenced by using the cBot and the HiSeq2000 from Illumina for single end reads with a base pair length of 50.

The RC dataset is accessible through GEO Series accession number GSE99897 for the RNA-Seq dataset and GSE100110 for the microarray data.

### Microarray data preprocessing and analysis

All preprocessing and statistical analyses of microarray data were performed using R statistical computing environment [19]. Affymetrix BL2 data was processed using the custom CDF file (*hugene10st_Hs_ENTREZG)*, *getting the most complete gene meta data annotation for the affymetrix probe ids*. *Afterwards* the Robust Multi-array Average (RMA) algorithm was applied [20]. Adittional quality control metrics for BL2 can be found in the supplements (S1 File). Both datasets were log2 transformed and quantile normalized. In case of several probes corresponding to the same Ensembl gene identifier, the probe with median expression intensities was chosen to represent the gene level expression. Differential expression analysis was performed by fitting linear models using empirical Bayes method as implemented in the limma r-package [21] and p-values were adjusted for multiple testing using Benjamini-Hochberg (BH) method [22].

### NGS data preprocessing and analysis

#### NGS quality control

The raw reads from both datasets were quality assessed using fastqc (http://www.bioinformatics.babraham.ac.uk/projects/fastqc/). Beside an agglomeration of nucleotides with slightly lower quality at the starting positions than in the middle of reads, no major quality issues were observed (S1 Table, S1a and S1b Fig). For each samples the distribution of unique, multi- and unmapped reads were checked for high proportion of unmapped or multi mapped reads, which were not explainable by the underlying alignment methods (S1 Appendix).

#### Generation of alignments

Different state-of-the-art RNA-Seq aligners were compared: STAR, TopHat2 and Sailfish.

STAR (v2.4.0h) is a splice-aware ultrafast universal RNA-Seq aligner, which utilizes a sequential maximum mappable seed search in uncompressed suffix arrays followed by seed clustering and a stitching procedure [23]. TopHat2 (v2.0.13) is as well a splice-aware RNA-Seq aligner which uses a two-step approach: 1. detecting potential splice sites for introns, 2. using these candidate splice sites in a subsequent step to correctly align multi exon-spanning reads [24]. Sailfish (v0.6.3) works differently and is not directly an aligner, since it avoids mapping of reads entirely and utilizes the observation of k-mers occurring in reads instead of alignments of reads [25].

Reads obtained from RNA sequencing were mapped against the reference genome of Homo sapiens Ensembl Version GRCh38.76 utilizing further information from the gene transfer format (.gtf) annotation from Ensembl version GRCh38.76. In case of Sailfish, it required a precomputed set of transcripts in fasta format. This was done with RSEM’s rsem-prepare-reference function providing the reference and the .gtf annotation.

#### Generation of counts

Multiple tools for counting of reads overlapping gene features were utilized: HTSeq, RSEM, Sailfish, and Cufflinks.

HTSeq-Count is a tool from the Python Toolbox HTSeq (v0.5.4p1) for counting reads overlapping into a specific feature (gene) [26]. RSEM (v1.2.19) is a software package for quantification of gene and isoform abundance estimation, utilizing an expectation maximization algorithm [27]. Sailfish an alignment-free tool to estimate isoform abundances via an expectation maximization algorithm, directly from a set of reference sequences, using k-mers as main transcript coverage unit. Cufflinks (v2.0.13) performs estimation of abundance with a likelihood based approach for simultaneous estimation of bias parameters and expression levels [28].

Later comparisons are based on tpm (transcript per million) values. Therefore, after statistical testing, the fragments per kilobase per million (fpkm) values and normal read count data were transformed (Supplement S2 Appendix) to tpm values for comparability in figures, using the R programming language.

#### Correlation analysis

The correlation analysis were done by taking the mean of each sample wise correlation test between pipeline methods. As distance measure we took 1-Pearson correlation, followed by complete linkage hierarchical clustering of the samples. All calculation were performed in R.

#### Analysis of differential gene expression

After counting reads, all abundance values were compared with edgeR, performing a likelihood ratio test (glmLRT). This R-package implements a range of statistical methods based on the negative binomial distributions, like empirical Bayes estimation, exact tests, generalized linear models and quasi-likelihood tests [29]. Cufflinks does not deliver read count data and therefore had to be tested by Cufflinks cuffDiff [30].

As cutoff for significantly differentially expressed genes after multiple testing correction (BH), a false discovery rate (FDR) of five percent was used. All results after differential gene expression were transformed into tpm (S2 Appendix) and the significant genes of each Pipeline result were used as input for gene ontology enrichment analysis.

#### Pipelines

**Fig 1:**
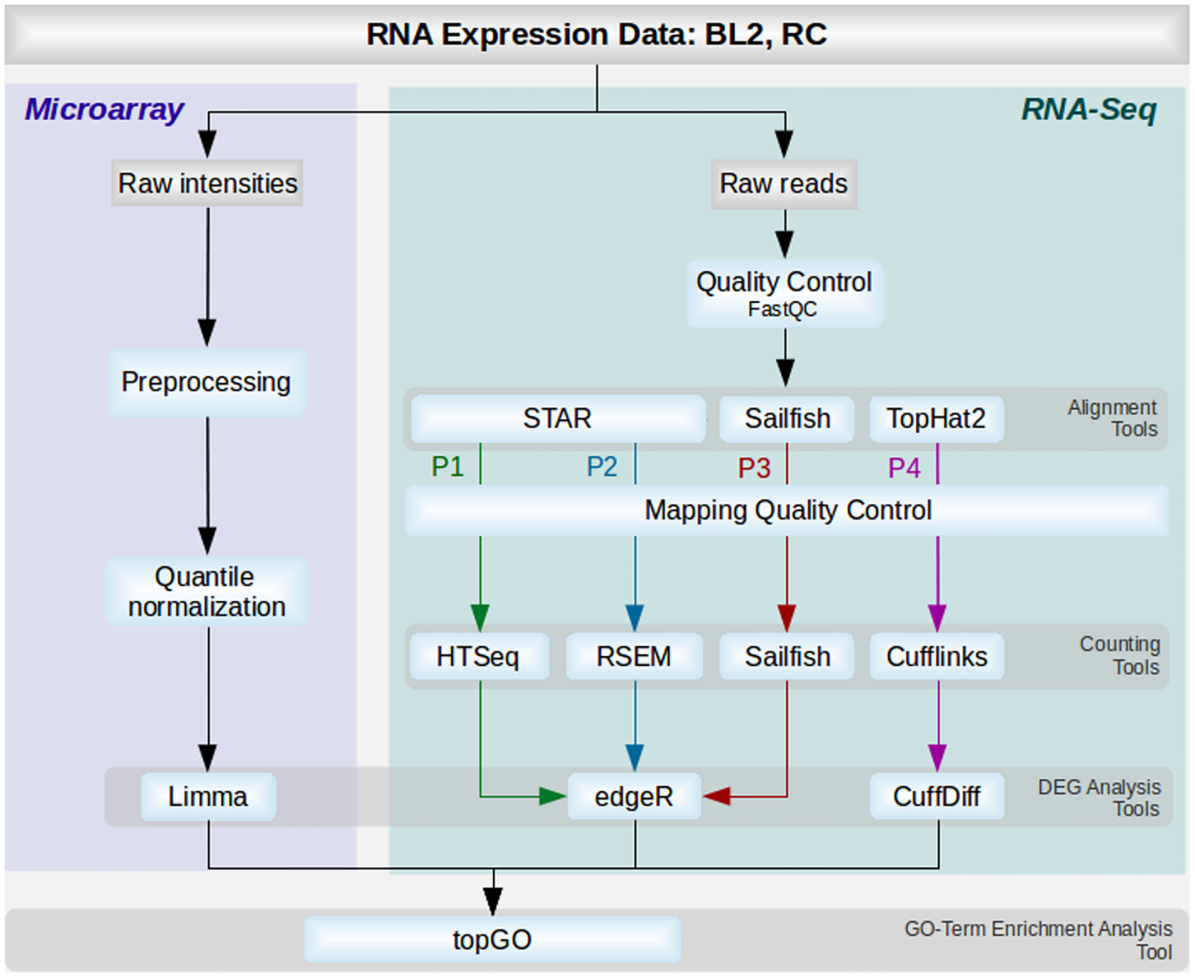
The different analysis pipelines. The flowchart describes the different tools and steps used for microarray (blue) and RNA-Seq analysis (green). Tasks and tools used at different steps are colored in light blue. Tools corresponding to the same steps are grouped and colored as follows: green (P1 with STAR, HTSeq and EdgeR), blue (P2 with STAR, RSEM and edgeR), red (Sailfish and edgeR) and purple (TopHat2, Cufflinks and CuffDiff).

Based on the described steps and tools used, 4 different pipelines were set up (Fig 1) and named as follows: P1(HTSeq) including STAR, HTSeq-Count and edgeR; P2(RSEM) consisting of STAR, RSEM and edgeR; P3(Sail) with Sailfish and edgeR; P4(Cuff) consisting of TopHat2, Cufflinks and CuffDiff.

#### Gene ontology enrichment analysis (GO analysis)

Genes with an FDR smaller than 5% where selected and sorted as a gene-list in ascending order. These gene-lists derived from edgeR, CuffDiff and limma were used as input for the weighted fisher-exact test implemented in the package TopGO version 1.0 [31] to calculate the enrichment for each GO-category. The GO-Enrichment analysis tests if a selected feature set (gene-list of DEGs) falls into a Gene Ontology category more often than expected by chance. GO-terms with a p-value smaller than 5% were considered significant and used subsequently for visualizations.

## Results and discussion

The aim of this study was to evaluate common analysis methods for RNA-Seq differential gene expression and cross-compare them with well established analysis methods for microarray. The comparisons were evaluated based on matched microarray and RNA-Seq profiles of two datasets: 1.) rectal cancer patient dataset comprising four patients with a good prognosis and six patients with a bad prognosis (referred to as RC dataset). 2.) Burkitt Lymphoma cell line dataset comprising three replicates of control cell line and three replicates of cell line stimulated with BAFF (referred to as BL2 dataset). RNA-Seq data was quality checked, aligned, qualities of mapped reads were manually investigated, reads were counted and analyzed for differential gene expression. The microarray data was preprocessed, quantile normalized and differentially expressed genes were detected. Finally a GO-term enrichment analysis was performed on the results of all used pipelines (Fig 1).

We evaluated four RNA-Seq pipelines (P1 –P4) based on different analysis steps: aligning (section ‘Performance of Alignment tools’), then we cross-compared these pipeline and microarray results (MA) based on correlation of expression levels (section ‘Gene-wise correlation of RNA-Seq and microarray data’), differential gene detection (section ‘Results of differential gene expression’) and pathway enrichment detection (section ‘GO-Enrichment analysis’).

### Performance of alignment tools

The BL2 data was profiled by poly-A-mRNA sequencing whereas RC data by total-RNA sequencing. For investigating the mapping performance on BL2 and RC, three different aligners for read mapping, STAR, TopHat2 and Sailfish, were investigated and the results compared.

The three aligners were evaluated based on their total mapping rate (see Table 1), where the aim should be to always map as much data correctly as possible. In the BL2 datasets the proportion of mapping rate results were close together. For the RC data, aligners that map to the genome (TopHat2, STAR) performed much better than an aligner mapping to a transcriptome, like Sailfish.

**Table 1:** Overview of total mapping rates over all samples in % for the different RNA-Seq aligner. Displayed are the mean mapping rates over the complete dataset with the variance in brackets.

Dataset\Tools	STAR	TopHat2	Sailfish
**BL2**	98.98 (±0.05)	97.02 (±0.1)	84.81 (±0.85)
**RC**	98.49 (±0.35)	96.73 (±0.4)	54.44 (±3.71)

**Fig 2:**
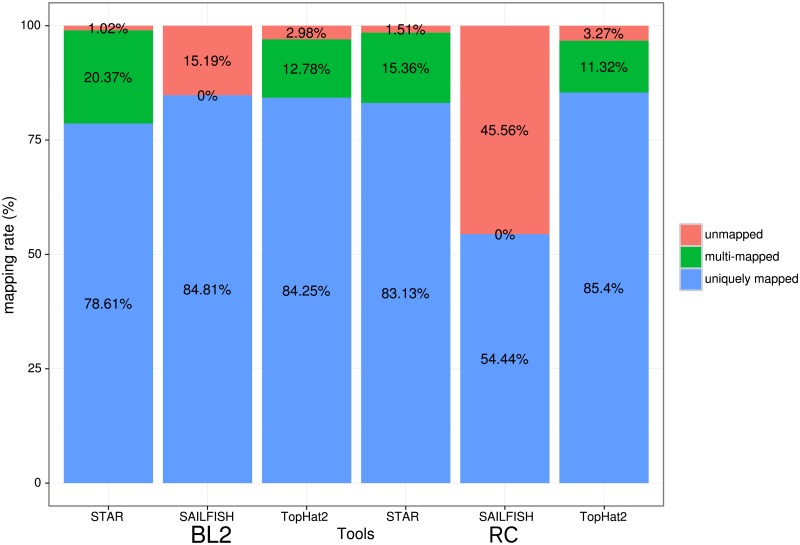
Mapping distribution. Mapping distribution in % for all three Aligners for both datasets. On the left the BL2 dataset and on the right the RC dataset is shown.

Therefore we took a closer look into the proportions of unique and multi mapping rates, which together result in the overall mapping rate (Fig 2). STAR showed an overall lower unique mapping rate (BL2 78.61%, RC 83.13%) than TopHat2 (BL2 84.81%, RC 85.4%), but got a higher total mapping rate of reads, due to a higher multimapping rate (STAR (BL2 20.37%, RC 15.36%), TopHat2 (BL2 12.78%, RC 11.32%)). For a full list of the complete mapping-performance see S1 Appendix. Depending whether or not to use multi mapped reads for later counting of features, in case of using them STAR performs slightly better than TopHat2. If only unique mapped reads are utilized, Sailfish performed the best for the BL2 dataset, which is based on poly-A mRNA sequencing. The performance of Sailfish on the RC dataset is a lot worse than for STAR or TopHat2. This is due to multiple reasons: as Sailfish is mapping against known transcripts only, its performance is based on the quality of the species reference transcriptome. For the BL2 dataset the unique mapping rate is 84.81%, which is better than STAR’s (78.61%) and TopHat’s unique mapping rate (84.25%). Sailfish allows only for perfect (unique) matched reads (kmers) and all multi mapped reads are inherently discarded during the processing by Sailfish. Variant rich data, contradicts the unique matching dogma of Sailfish, which could lead to less mapped reads overall. This phenomenon described is illustrated in the RC data by only 54.44% of mapped reads. The data is based on total-RNA sequencing, where only around 54% of the dataset is annotated when building the transcriptome, as such a large proportion of it seems still unknown and therefore cannot be mapped with Sailfish, yet.

Nevertheless, in terms of time required for aligning the data, the performance of Sailfish was the fastest. As an example: the read mapping for RC took Sailfish 6–7 minutes per dataset including the counting step, whereas STAR took 6–10 minutes per aligning sample and TopHat2 up to three hours.

### Evaluation of RNA-Seq pipelines and cross-comparison with microarray

#### Gene-wise correlation of RNA-Seq and microarray data

We performed a gene-wise correlation analysis based on expression levels after counting. We correlated the different quantification levels after they were transformed into log2 tpm (RNA-Seq) and log2 quantile normalized expression values (microarray).

**Fig 3:**
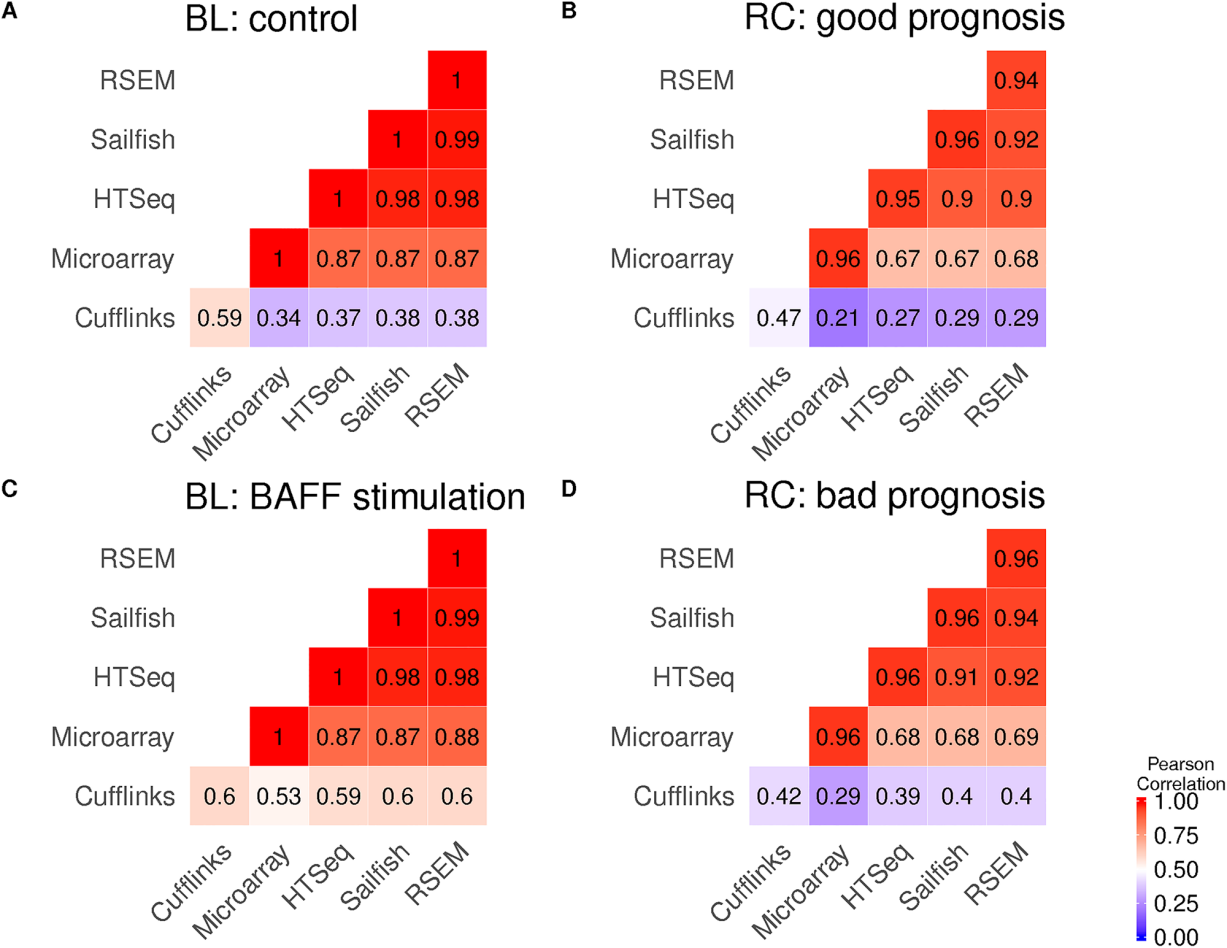
Correlation of all samples after analysis. The heatmap describes the combined Pearson correlation coefficient over all pairwise correlation tests of normalized gene expression against all replicates between groups. For RNA-Seq all expression values are normalized to tpm (transcript per million), to be able to compare them. Fig. 3A and 3C show the BL2 dataset and the correlation of all samples before (A) and after (C) BAFF stimulation for each analysis tool used. Fig 2B and 2D show the correlation of patient samples for the two groups with good prognosis of distant free metastases (good) and a bad prognosis (bad) together with the different analysis pipelines used.

The correlation heatmaps shown in Fig 3 were done by taking the mean of each sample wise correlation test between pipeline methods. Last, they were clustered based on complete linkage with the distance 1-Pearson correlation. Overall we observed a high correlation on all performed RNA-Seq Pipeline runs together with the corresponding microarray values, which was observed similarly in other studies as well [32] [33] [34] [35] for different datasets. The correlation of microarray and RNA-Seq data is moderately worse for the RC data (ranges of 0.67 to 0.68) than for the BL2 data (0.87 to 0.88), which can be expected, since the overall biological variability of patient data is higher than in cell lines. The overall difference in correlation between microarray and RNA-Seq can be explained by their technological difference in the quantification of the gene expression. For RNA-Seq analysis the Pipeline P4 utilizing Cufflinks and CuffDiff was a big surprise since the mean correlation coefficients where quite low, even when correlating the replicate of the same method with each other. Microarray methods measure the intensities of fluorescence, which mirrors the associated gene expression, whereas RNA-Seq methods measure read counts as associated relative abundance measure for gene expression levels. Interestingly, the correlation between the different RNA-Seq tools is high (BL2: 0.97 to 0.99, RC: 0.94 to 0.98). Only a minor impact of mapping and counting approaches is observed in correlation coefficients. RSEM shows the highest correlation with the microarray data on both sets closely followed by Sailfish, HTSeq-Count and Cufflinks.

#### Results of differential gene expression

On the differential gene expression level all pipelines were compared based on the number of significantly differentially expressed genes (DEGs). P1, P2 and P3 were tested for differential expression with edgeR, P4 with the CuffDiff script of Cufflinks and limma was used for microarray analysis.

Overall the number of DEGs for the RNA-Seq pipelines were much higher than observed for the microarray analysis. Moreover, we evaluated overlaps of DEGs between the P1-P4 pipelines. In particular, we focused on the subsets of genes that were detected by at least two out of four pipelines ('consensus DEGs') and the subset of genes that were detected solely by only one pipeline ('DEGs unique'). In the absence of the ‘ground truth’ we use these measures (‘consensus DEGs’ and ‘unique DEGs’) as indicator of pipeline performance in terms of identifying potential true-positive results and false-positive results. Also these proportion are only indicators and are by no means actual true-positives and false-negatives, but more reflecting the general agreement with the other compared methods and the inconsistency or uniqueness of each individual method.

#### Microarray results

For the two investigated datasets we identified very low numbers of DEGs using the microarray platform. For BL2, out of 1196 genes significant on p-value level only 1 gene (*GPER*) remained significant after FDR correction. For the RC dataset, out of 1285 genes significant on p-value level no gene remained significant after FDR correction (S2b Fig). We checked these genes with the results of differential expression analysis for microarray reported in Schrader et al. [18], which did a similar study with nearly the same condition. We could reidentify 26 genes based on the gene symbols in common (see supplement S2 Table). A reason for this small number of overlapping genes could be attributed to the difference in power of the analysis and experimental conditions. Our BL2 dataset was newly resequenced, incubated for 24h instead of 9h and the microarray chip used was the HG-U133_Plus_2 chip instead of U133 plus 2.0.

#### RNA-Seq Pipeline comparison on BL2

P1 to P3 found the highest amount of DEGs (287, 340, 375) after FDR correction. For P4, only 20 genes were left and for the microarray results one gene was left significant after p-value adjustment (Table 2). 29 of these FDR corrected genes could also be reidentified from a former study from Schrader et al. [18] and are commonly shared by the pipelines of P1, P2 and P3 (see supplement S2 Table). When adding P4, the number reduced to 4.

**Table 2:** Overview of the number genes and GO-terms significant (p-value <5%) and after FDR correction for P1-4 and microarray. The GO-terms for microarray are in bold, because the p-value was used as a cutoff instead of the FDR.

Pipelines	Number of DEGs (p-value)		Number of DEGs (FDR)		Number of sign. GO Terms	
	BL2	RC	BL2	RC	BL2	RC
**P1(HTSeq)**	2299	3377	287	71	138	127
**P2(RSEM)**	2329	3646	340	96	131	111
**P3(Sail)**	2410	1285	375	146	158	127
**P4(Cuff)**	316	1398	20	154	89	96
**Microarray**	1196	1289	**1**	**0**	**116**	**148**

**Fig 4:**
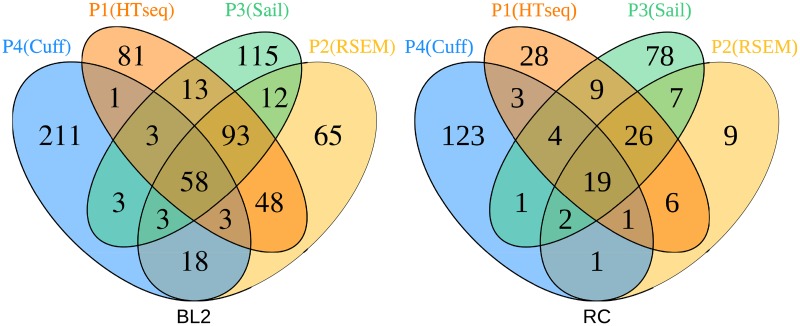
Significant overlapping genes for the different strategies after multiple test adjustment. Shown are two venn diagrams, one for each dataset (BL2 Fig. 4A and RC Fig. 4B). The different pipelines used here are: TopHat2 and Cufflinks (T&C), STAR and HTSeq-Count (S&HT), Sailfish (Sa), STAR and RSEM (S&R). The microarray data is not included, because there were close to no significant genes after FDR adjustment.

Next, we identified the different overlaps of DEGs for the individual pipelines (P1-4 in Fig 4 BL2). Since we don’t know the true calls for the dataset we utilized ‘consensus DEGs’ and ‘unique DEGs’ as surrogate measures supporting the interpretation of the overlaps.

Considering all four RNA-Seq pipelines a total of 9 genes can be found in common by all pipelines for BL2. P4(Cuff) pipeline detected 20 DEGs from which 9 were found also by the other pipelines. These genes are: *TSPAN11*, *PFKFB4*, *SGK1*, *CCR7*, *NFKBIE*, *CCDC28B*, *HLA-DQA1*, *HLA-DRB5*, *HLA-DQA2*.

For evaluation purposes, we consider the genes found by the majority of tools as promising candidates for true findings. Therefore, we are looking at the overall agreement between the tools in form of overlaps of genes in common by at least 2 other pipelines (Table 3) as a measurement of potentially true findings for the tools. It can be seen that P1(HTSeq) has the largest number of significant genes also found by others (211/287), which is 73.52% of their complete findings. P1 got the lowest percentage of genes found unique (19.16%), which translates to 55 out of 287 genes. Genes not found by other pipelines can either way be interpreted as false calls or as simply missed by the other pipelines or most likely a mixture of both. Since finding false calls in general is not desired, we tend to consider a low amount of unique genes found as positive. Following this interpretation, the quality of the pipelines in their outcome for the BL2 dataset can be ordered as follows: P1(HTSeq), P2(RSEM), P3(Sail), P4(Cuff).

**Table 3:** Overview of the proportion of genes and corresponding percentage of differential expressed genes for each pipeline after multiple testing adjustment. ‘consensus’ stands for the amount of genes shared with at least two other pipelines and ‘unique’ for genes not found by any other Pipeline from the total amount of genes found by each Pipeline.

Pipelines	Consensus DEGs				DEGs unique			
	BL2		RC		BL2		RC	
**P1(HTSeq)**	73.52%	(211/287)	67.60%	(48/71)	19.16%	(55/287)	12.68%	(9/71)
**P2(RSEM)**	49.70%	(169/340)	52.08%	(50/96)	29.41%	(100/340)	29.17%	(28/96)
**P3(Sail)**	52.80%	(198/375)	34.93%	(51/146)	41.60%	(156/375)	53.42%	(78/146)
**P4(Cuff)**	45.00%	(9/20)	16.88%	(26/154)	55.00%	(11/20)	79.87%	(123/154)

#### RNA-Seq pipeline comparison on RC

The ordering of the highest amount of significant genes after FDR correction flipped in this dataset for P1-4. This time P4 found the largest number of significant genes (154), followed by P3(146), P2(96) and P1(71). P1 has the highest percentage of consensus DEGs 67.60% (48/71), followed by P2 52.08% (50/96), P3 34.93% (51/146) and P4 16.88% (26/154) (Table 3). A total of 19 genes (Fig 4 RC) can be found in common, namely: *EYA1*, *NPR3*, *MUC5B*, *RSAD2*, *IGF2BP3*, *ITGA11*, *IFI44L*, *IFI44*, *ASZ1*, *MX1*, *CTHRC1*, *FAM3B*, *POU5F1B*, *COL11A1*, *C1QC*, *SLC35D3*, *ZFHX4*, *MMP11*, *ANO1*. P4 has the highest number of DEGs, but only 26 of them found by others, whereas a total of 79.87% (123/154) of the genes cannot be found by any of the other pipelines.

This is highest rate of unique genes found, as well as the lowest rate of Consensus DEGs consistent in both datasets, despite here having the most genes found. Notably, Cufflinks is coupled with CuffDiff for the differential expression analysis, so the results of Cufflinks are as well influenced by differences in the statistical analysis. Overall P4 is the most divergent from all others, whereas P1 provides the results most concordant with the other pipelines, followed by P2.

#### GO-Enrichment analysis

**Fig 5:**
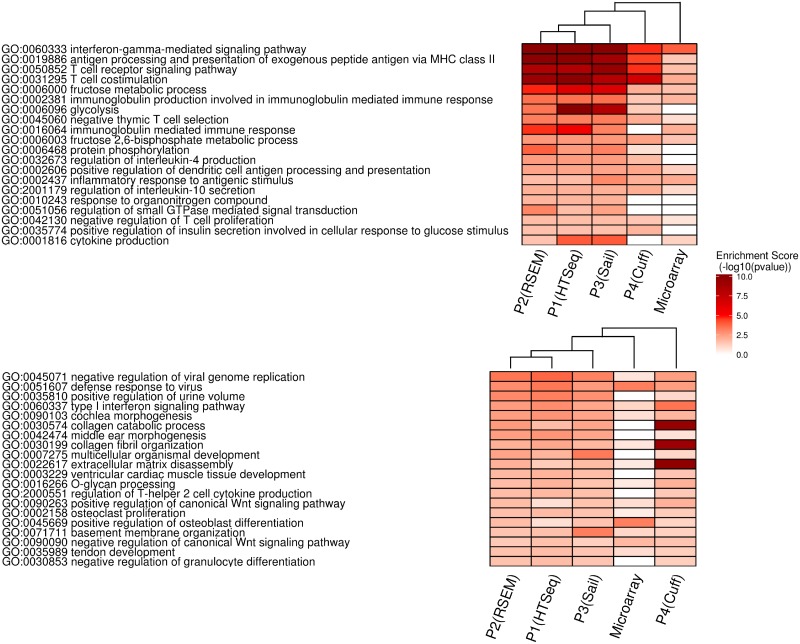
Top20 significant enriched GO-categories for BL2 and RC. In all shown RNA-Seq and microarray strategies the visualized GO-categories were enriched (p-value smaller than five percent and the pathway was bigger than four genes). The enrichment of GO-terms is shown in red: the higher the intensity of red, the lower the p-value. For better scalability of colors the negative log 10 was chosen. The pathways agreeing the most amongst all pipelines are shown at the top.

To evaluate the results from BL2 and RC datasets after differential gene expression analysis enriched GO-terms were investigated. Microarray results showed close to no significant genes after FDR correction. To nevertheless generate GO-term enrichment results for microarray based datasets, their significance threshold for the enrichment test was altered to <5% of the p-value instead of <5% FDR value (Table 2). Fig 5 shows the top 20 significant GO-terms from the different pipelines and microarray datasets. Hierarchical clustering of all GO-terms was applied to investigate the similarity of the different pipelines based on the enrichment scores. The complete set of significant pathways is depicted in S3a Fig for dataset BL2 and in S3b Fig for dataset RC.

#### BL2 dataset

In the context of comparing control Burkitt Lymphoma cell-line with the BAFF stimulated BL2 cells, it is to be expected to detect GO-terms related to the immune response as significantly enriched [36].

We checked whether we find this biological context in the top 20 significantly enriched GO -terms. In Fig 5A we can observe four highly enriched GO-terms related to immune response (GO:0060333, GO:0019886, GO:0050852, GO:0031295), primarily for pipelines P1-P4. In total we see 13 out of the 20 GO-terms linked to the immune system. In addition, four of the depicted GO-terms are related to metabolism, two to cell signaling and the last one to biological regulation. The enriched terms based on the microarray data leads to only one GO-term being highly significant, while 7 were not detected to be significantly enriched at all. Fig 5a shows that P1 to P3 perform similarly in terms of additional enrichment analysis. However Pipeline P4 and the microarray datasets show highly different enrichment scores. In summary, the deregulated GO-terms associated to immune response are identified by the majority of the pipelines.

#### RC dataset

When comparing GO-term analysis results of the rectal cancer patient group, we expect to see GO-terms related to metastases formation, like increased proliferation, cell rearrangements, changes in cell organization and to a certain extent immune response as well. Therefore we checked again if we observe several of these assumptions within the top 20 significantly enriched GO-terms. In these we observed GO-terms (GO:0090263, GO:0002158, GO:0090090) linked to cellular proliferation, GO-terms (GO:0030199, GO:0022617, GO:0071711) linked to cellular rearrangements and GO-terms (GO:0060337, GO:2000551, GO:0030853) related to immune system response. However a lot of significant GO-terms could not be related to any of the expected cellular response classes. As previously discussed, these results might be due to sequencing of total-RNA in case of the RC dataset in comparison to polyA-mRNA sequencing. In addition, the biological variability of human patient data is much higher in contrast to cell line data. Based on the data processed by P4 three GO-terms were identified to be highly enriched (GO:0030574, GO:0030199, GO:0022617). These are linked to (extra-)cellular rearrangements and fit well into our expectations, however could only be detected with such high enrichment score based on P4. In comparison, P1-P3 look very similar as the BL2 dataset.

## Conclusion

This study presents a comparison of RNA-Seq specific pipelines as well as a cross comparison with matched microarray data. For the investigated realistic datasets microarray analysis was inferior to the used RNA-Seq analysis strategies and only a minor proportion of DEGs already reported by Schrader et al. could reproduced. Pipelines P1 to P3 performed rather similar when looking at the correlation results, with a small lead in regard to utilization of raw data for P3. In contrast, P1 outperformed the rest in terms of the highest agreement with the other pipelines in the detection of differentially expressed genes. Results from P4 varied a lot, presumably due to the use of the internal Cufflinks statistics in the tool suite.

## Supporting information

S1 File Additional quality control metric for the BL2 and RC data sets. (GZ)

S1 Appendix Mean readmapping-rates and their standard-deviation. (DOC)

S2 Appendix R-functions for converting count and fpkm values into TPM. (DOC)

S1 Table Summary of RNA-Seq data. (DOC)

S2 Table Overview of genes shared in common between our results and DEG results reported by Schrader et al. (DOC)

S1 Fig Overview of qualities metrics for the BL2 (A) and RC (B) RNA-Seq data set. (PDF)

S2 Fig Overview of the amount of FDR corrected significant genes overlapping each pipeline and microarray for the BL2 (A) and RC (B) dataset. (PDF)

S3 Fig The complete list of GO-Terms for all 5 pipelines for the BL2 (A) and RC (B) dataset. (PDF)
