# Correction: A comparative study of RNA-Seq and microarray data analysis on the two examples of rectal-cancer patients and Burkitt Lymphoma cells

**DOI:** 10.1371/journal.pone.0224062

**Published:** 2019-10-24

**Authors:** Alexander Wolff, Michaela Bayerlová, Jochen Gaedcke, Dieter Kube, Tim Beißbarth

In the RNA-Seq Pipeline comparison on BL2 subsection of the Evaluation of RNA-Seq pipelines and cross-comparison with microarray subsection of the Results and discussion, there is an error in the third sentence of the fourth paragraph. The correct sentence is: It can be seen that P1(HTSeq) has the largest number of significant genes also found by others (169/287), which is 58.89% of the complete findings.

In Table 3, there are errors in the BL2 values of the Consensus DEGs column. Please see the correct Table 3 here.

**Table 3:** Overview of the proportion of genes and corresponding percentage of differential expressed genes for each pipeline after multiple testing adjustment.

Pipelines	Consensus DEGs				DEGs unique			
	BL2		RC		BL2		RC	
**P1(HTSeq)**	58.89%	(169/287)	67.60%	(48/71)	19.16%	(55/287)	12.68%	(9/71)
**P2(RSEM)**	49.70%	(169/340)	52.08%	(50/96)	29.41%	(100/340)	29.17%	(28/96)
**P3(Sail)**	45.07%	(169/375)	34.93%	(51/146)	41.60%	(156/375)	53.42%	(78/146)
**P4(Cuff)**	45.00%	(9/20)	16.88%	(26/154)	55.00%	(11/20)	79.87%	(123/154)

‘Consensus’ stands for the amount of genes shared with at least two other Pipelines and ‘unique’ for genes not found by any other Pipeline from the total amount of genes found by each Pipeline.

In Fig 4, there are errors in the labeling as well as the BL2 values. Please see the correct Fig 4 here.

**Fig 4:**
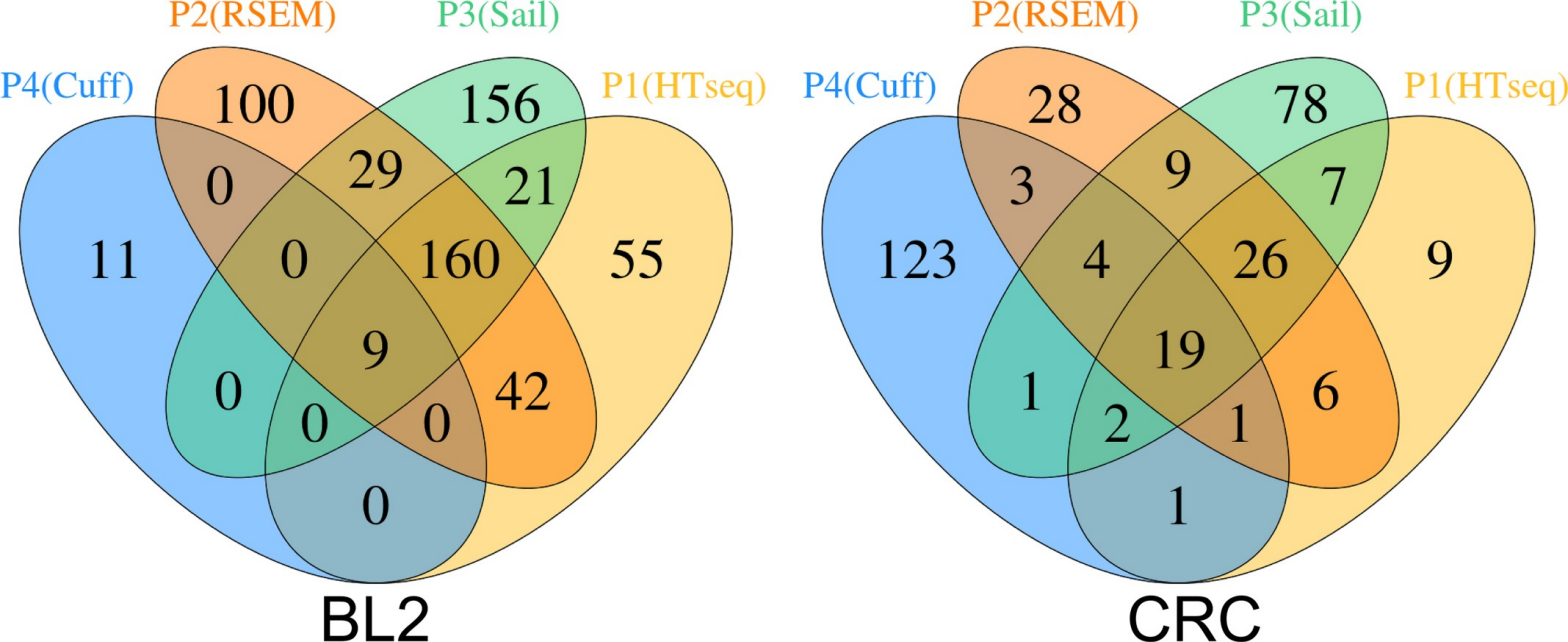
Significant overlapping genes for the different strategies after multiple test adjustment. Shown are two Venn diagrams, one for each dataset (BL2 Fig 4A and RC Fig 4B). The different pipelines used here are: TopHat2 and Cufflinks (T&C), STAR and HTSeq-Count (S&HT), Sailfish (Sa), STAR and RSEM (S&R). The microarray data is not included, because there were close to no significant genes after FDR adjustment.
