# Supplementary figures and images for: A comparative study of RNA-Seq and microarray data analysis on the two examples of rectal-cancer patients and Burkitt Lymphoma cells
Source: PLoS One. 2018 May 16;13(5):e0197162. doi: 10.1371/journal.pone.0197162 (PMC5955523; doi:10.1371/journal.pone.0197162)

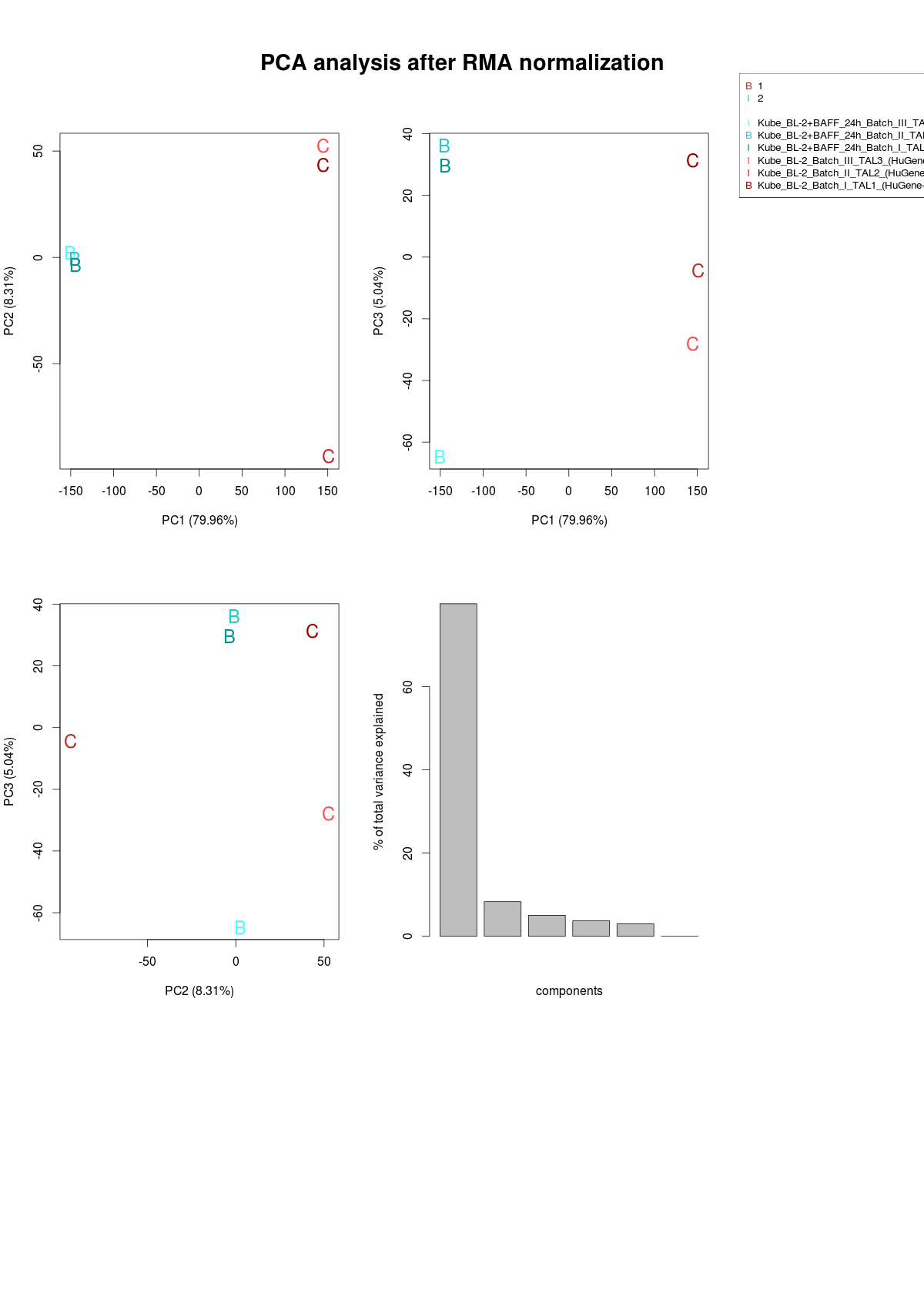

Supplement: S1 File — (GZ) [file pone.0197162.s001.tar.gz › NormDataPCAanalysis.png]

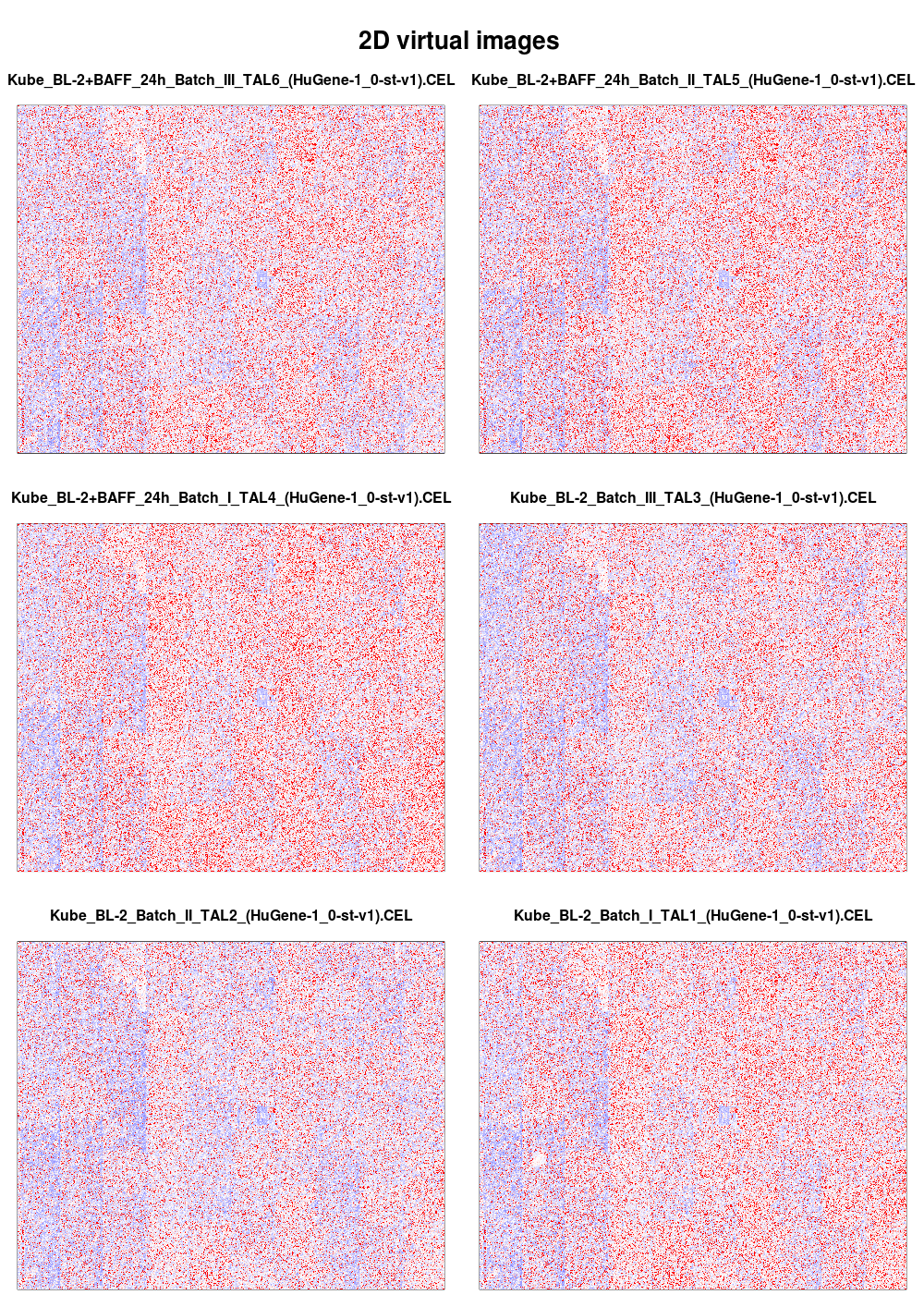

Supplement: S1 File — (GZ) [file pone.0197162.s001.tar.gz › RawDataArray.image.png]

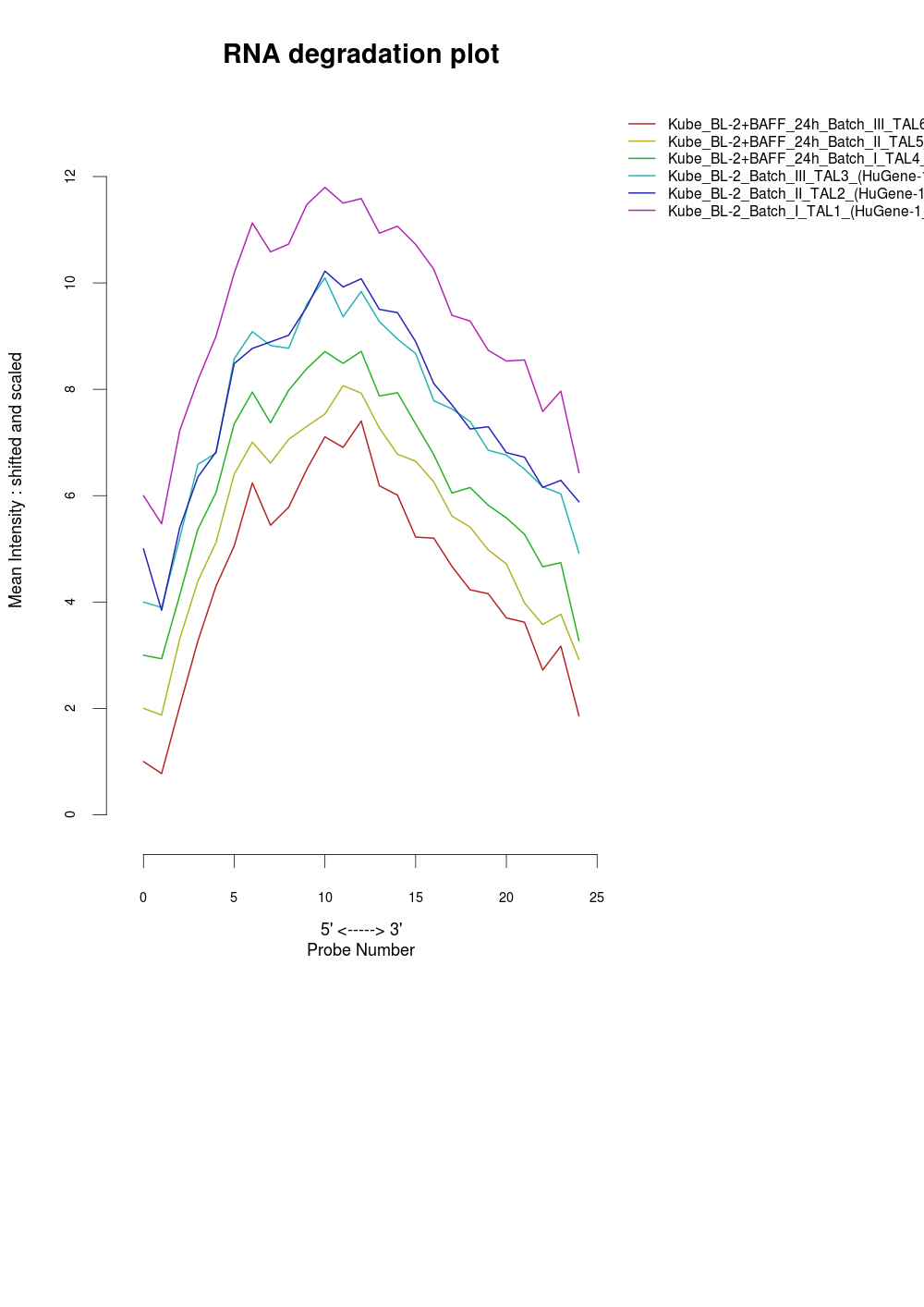

Supplement: S1 File — (GZ) [file pone.0197162.s001.tar.gz › RawDataRNAdegradation.png]

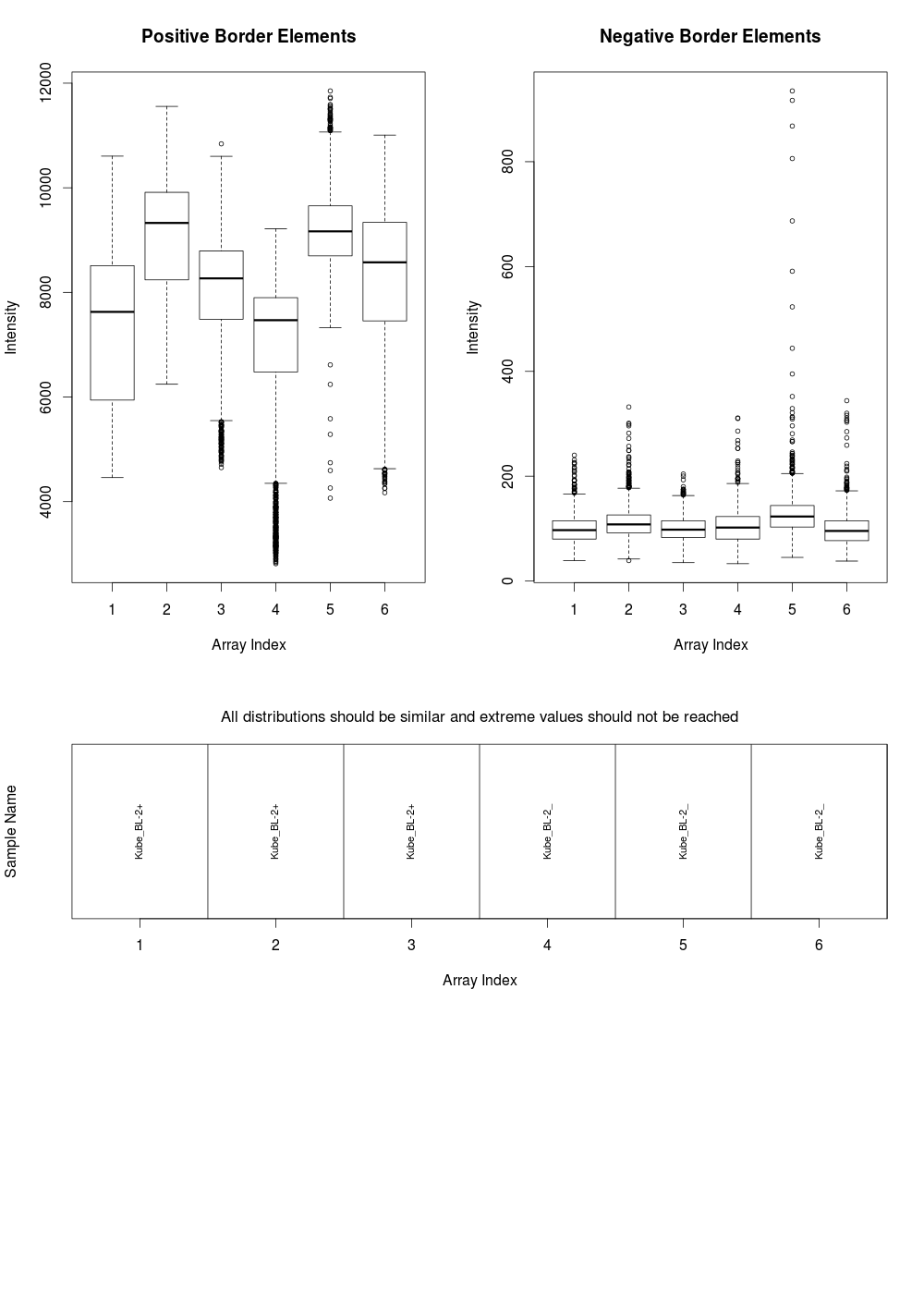

Supplement: S1 File — (GZ) [file pone.0197162.s001.tar.gz › RawDataPosNegDistribution.png]

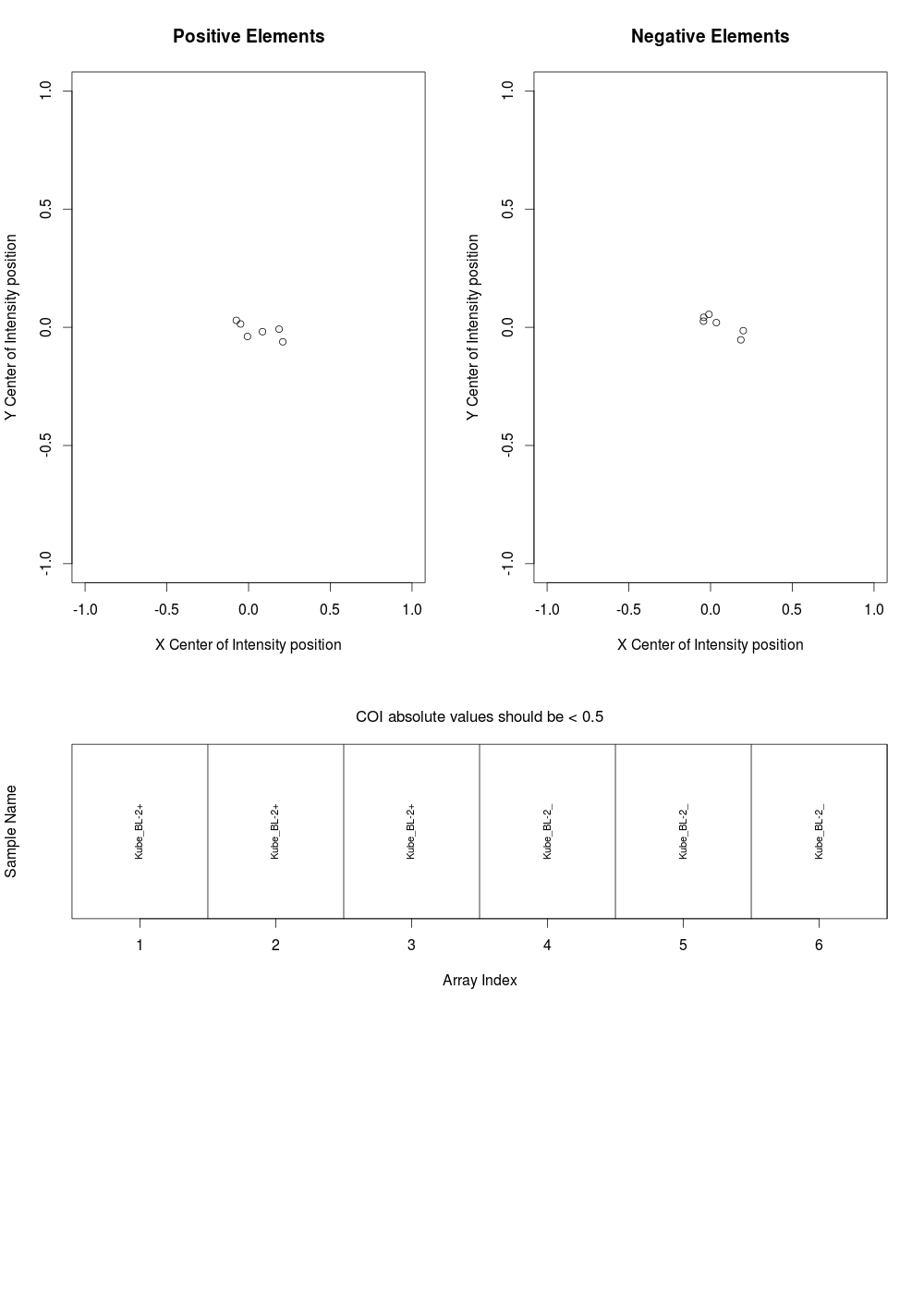

Supplement: S1 File — (GZ) [file pone.0197162.s001.tar.gz › RawDataPosNegPositions.png]

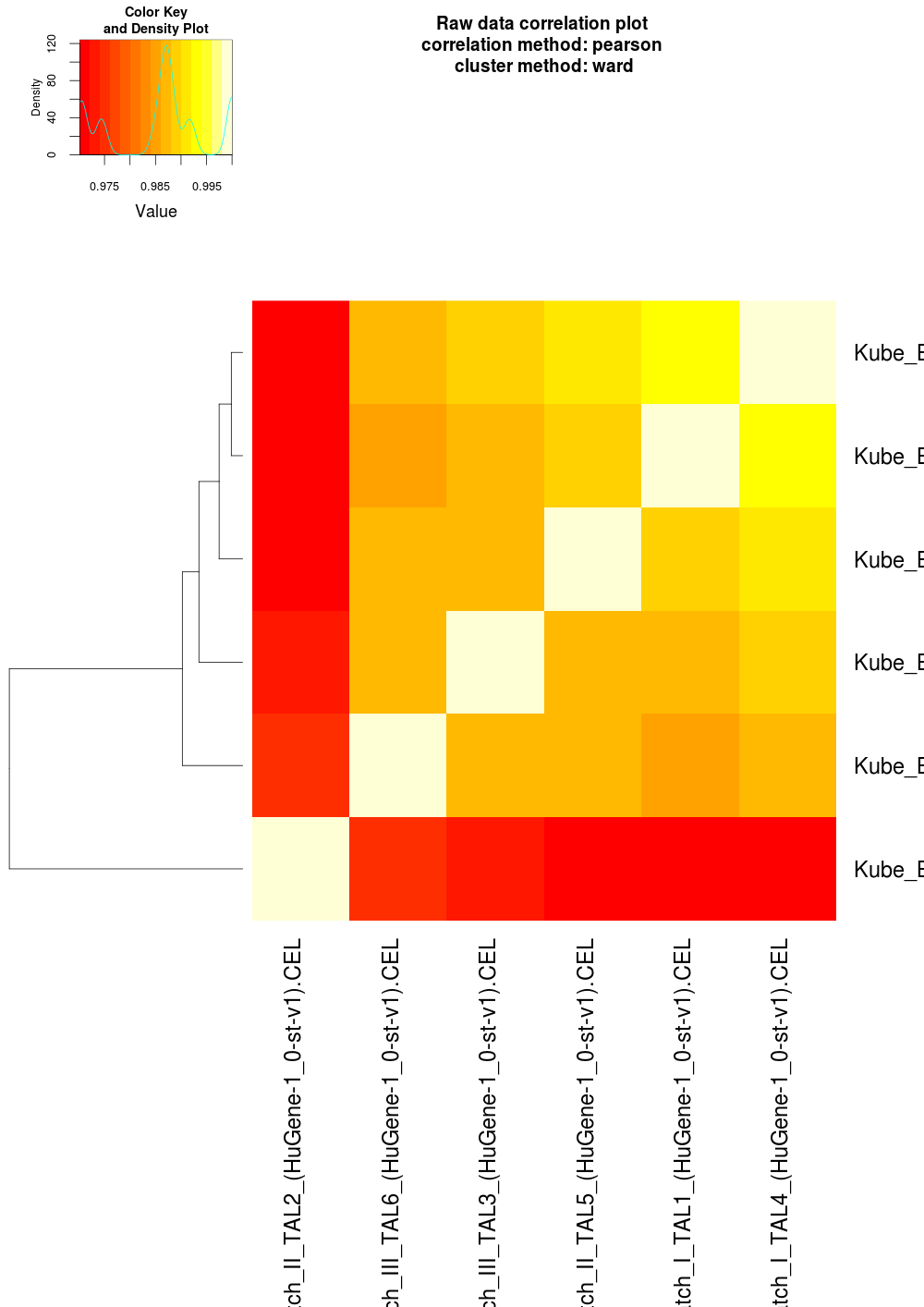

Supplement: S1 File — (GZ) [file pone.0197162.s001.tar.gz › RawDataArrayCorrelation.png]

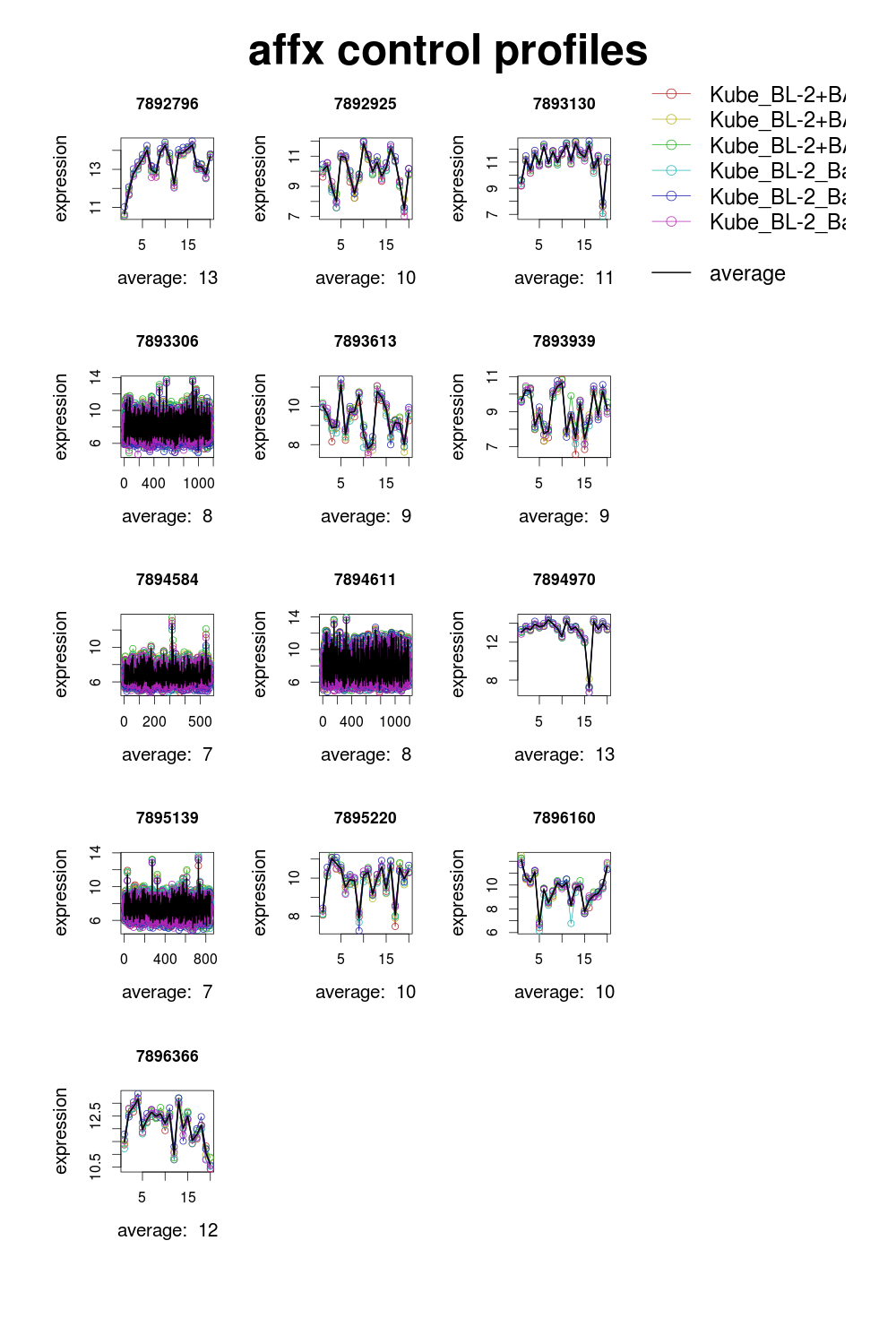

Supplement: S1 File — (GZ) [file pone.0197162.s001.tar.gz › RawDataAFFXControlsProfiles.png]

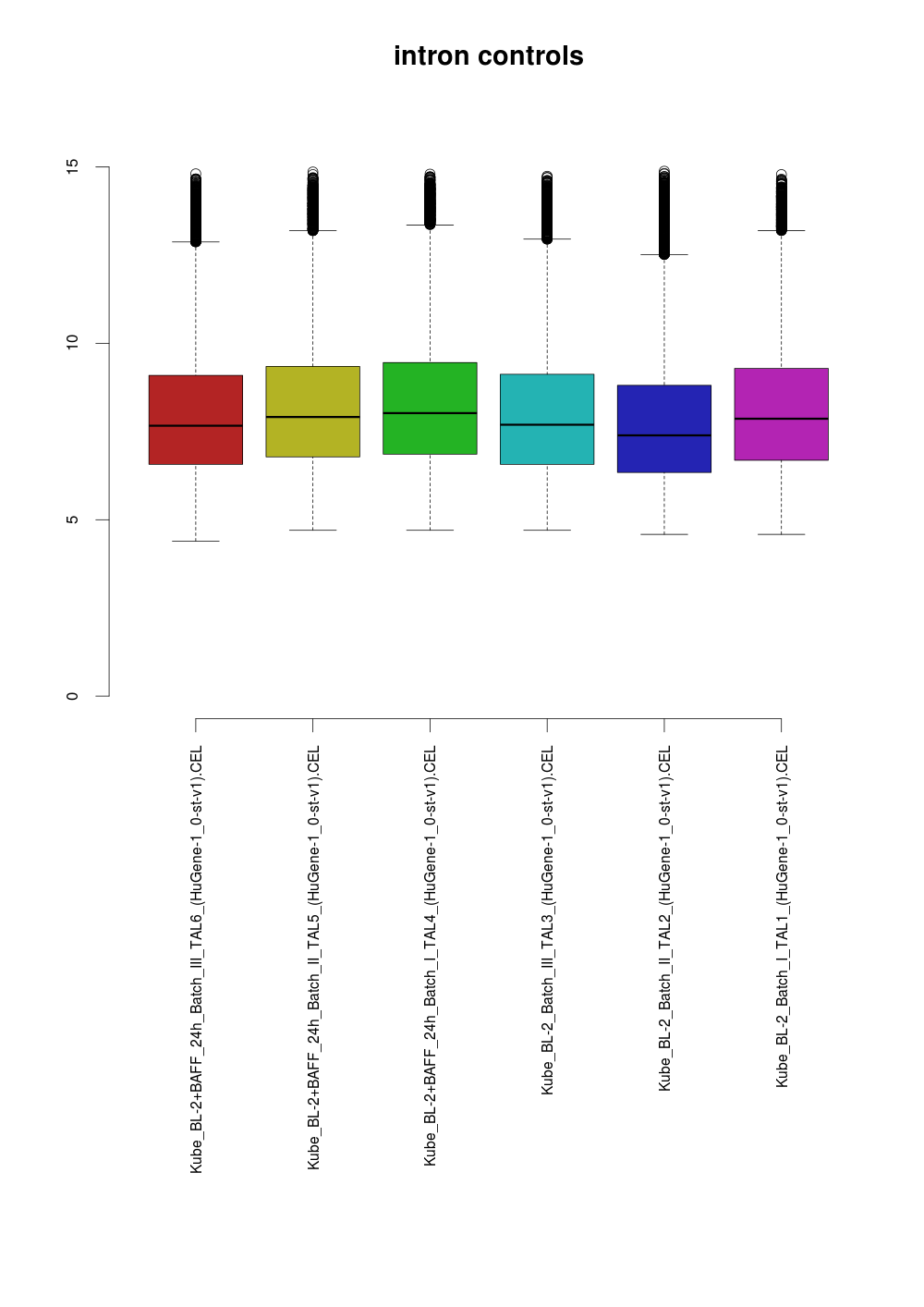

Supplement: S1 File — (GZ) [file pone.0197162.s001.tar.gz › RawDataINTRONControlsBoxplot.png]

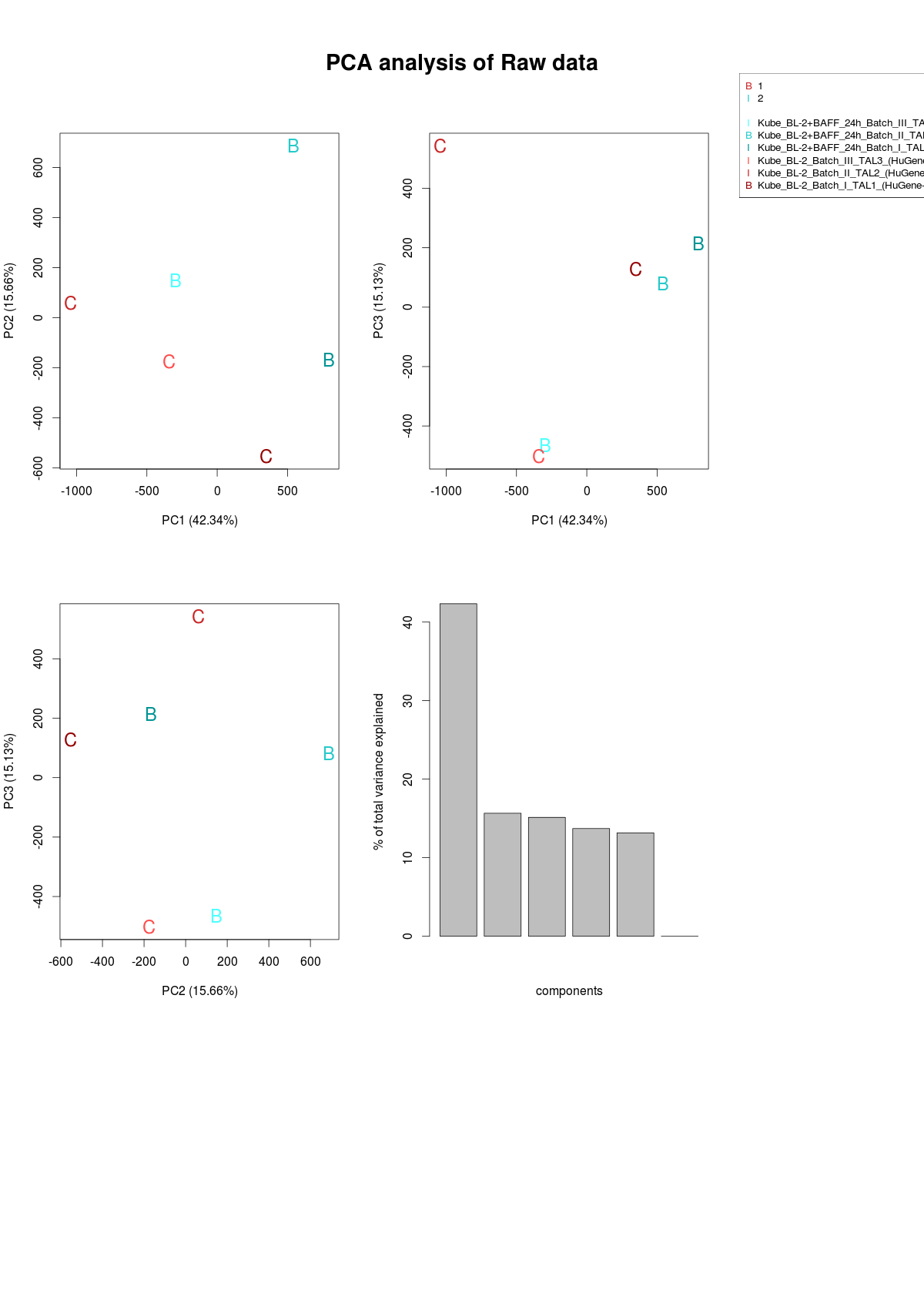

Supplement: S1 File — (GZ) [file pone.0197162.s001.tar.gz › RawDataPCAanalysis.png]

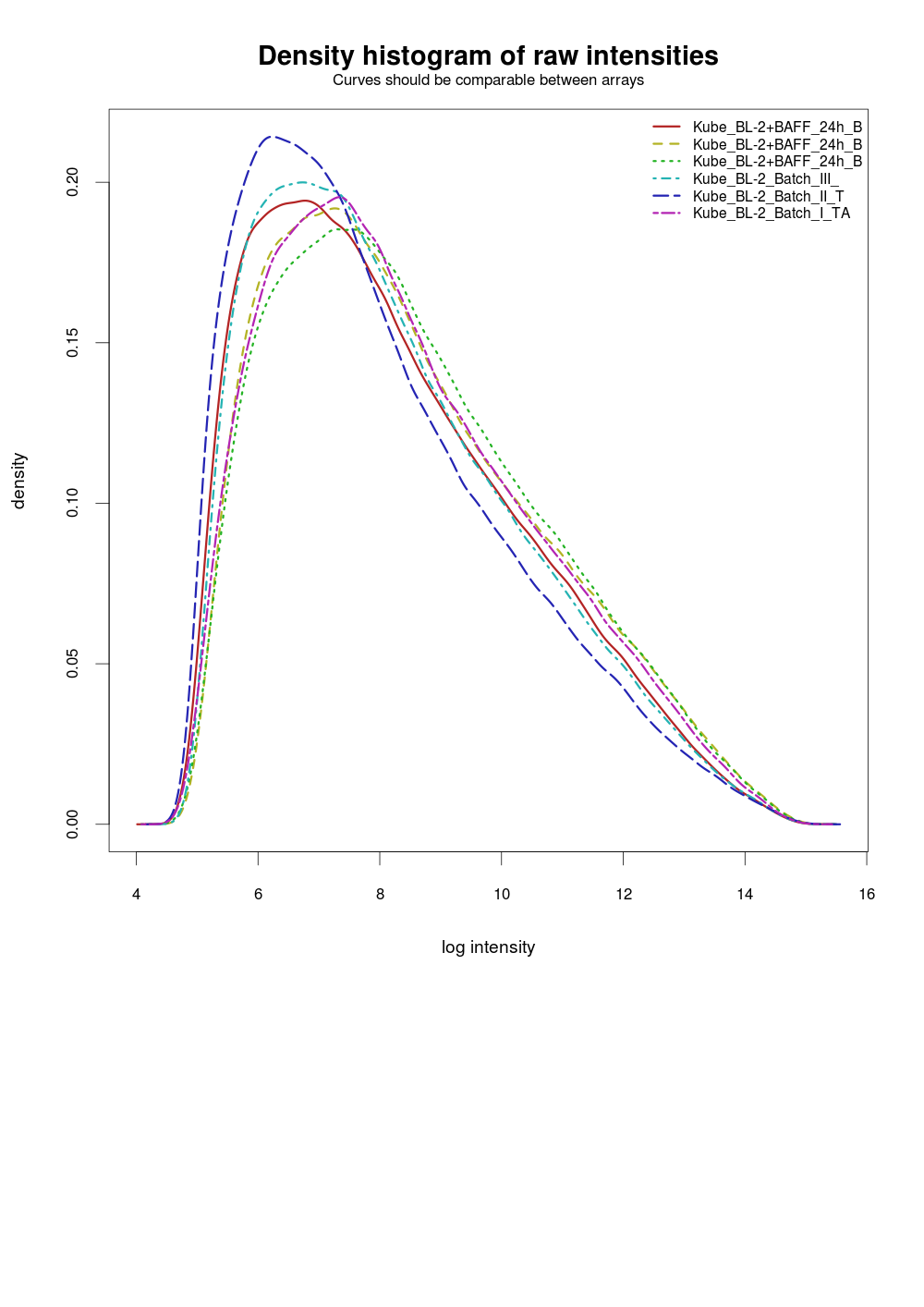

Supplement: S1 File — (GZ) [file pone.0197162.s001.tar.gz › RawDensityHistogram.png]

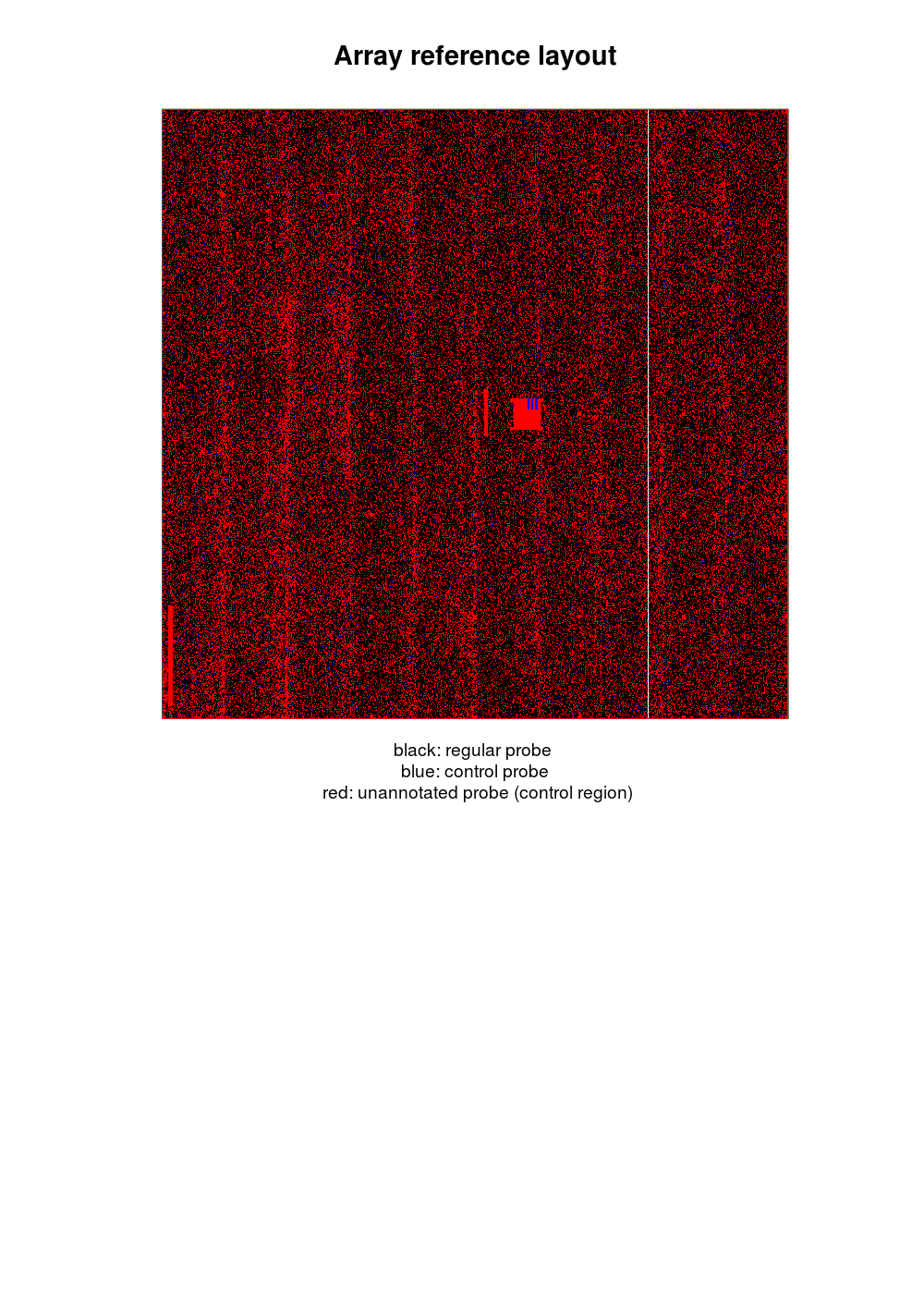

Supplement: S1 File — (GZ) [file pone.0197162.s001.tar.gz › RawDataReferenceArrayLayout.png]

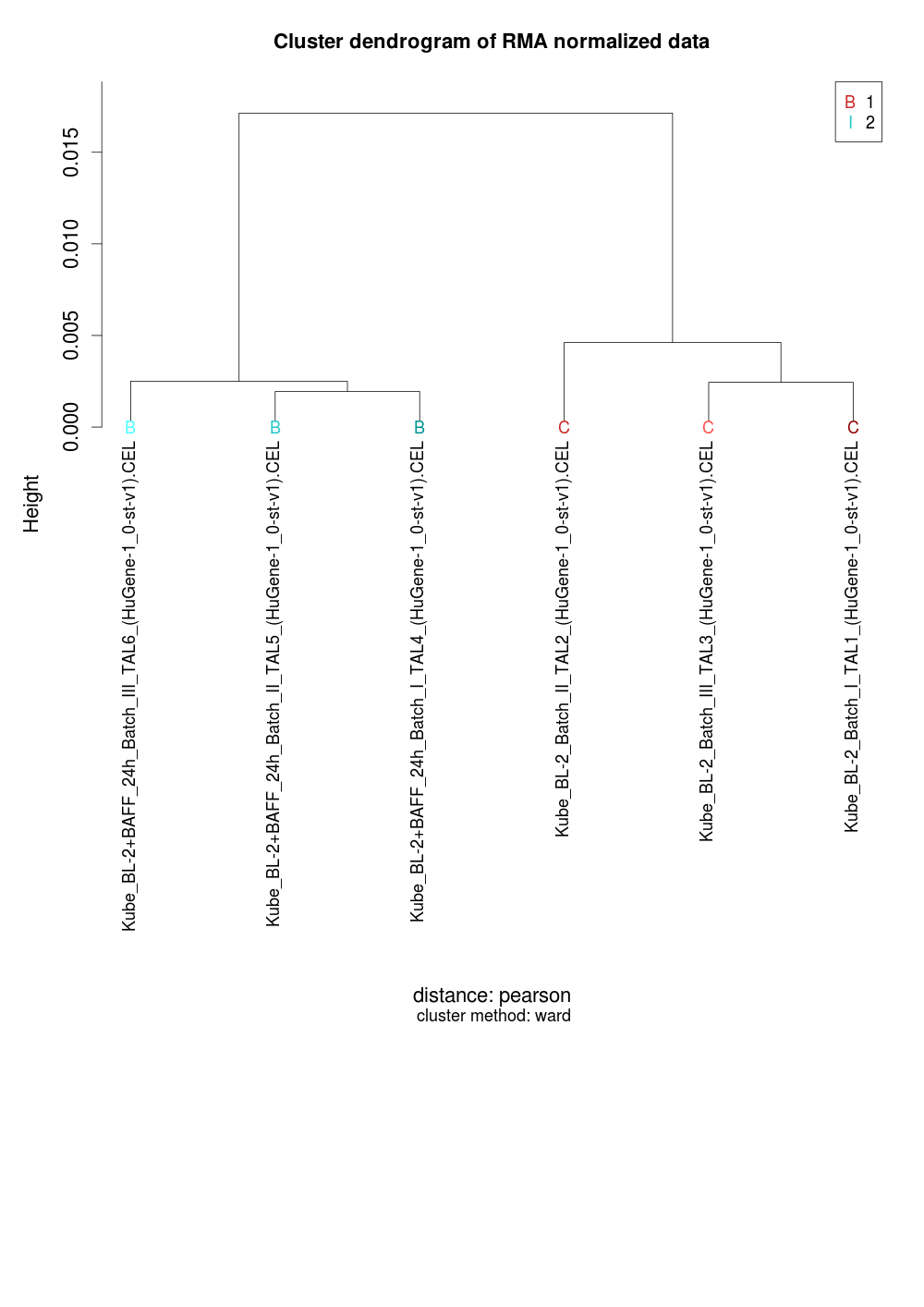

Supplement: S1 File — (GZ) [file pone.0197162.s001.tar.gz › NormDataCluster_pearson.png]

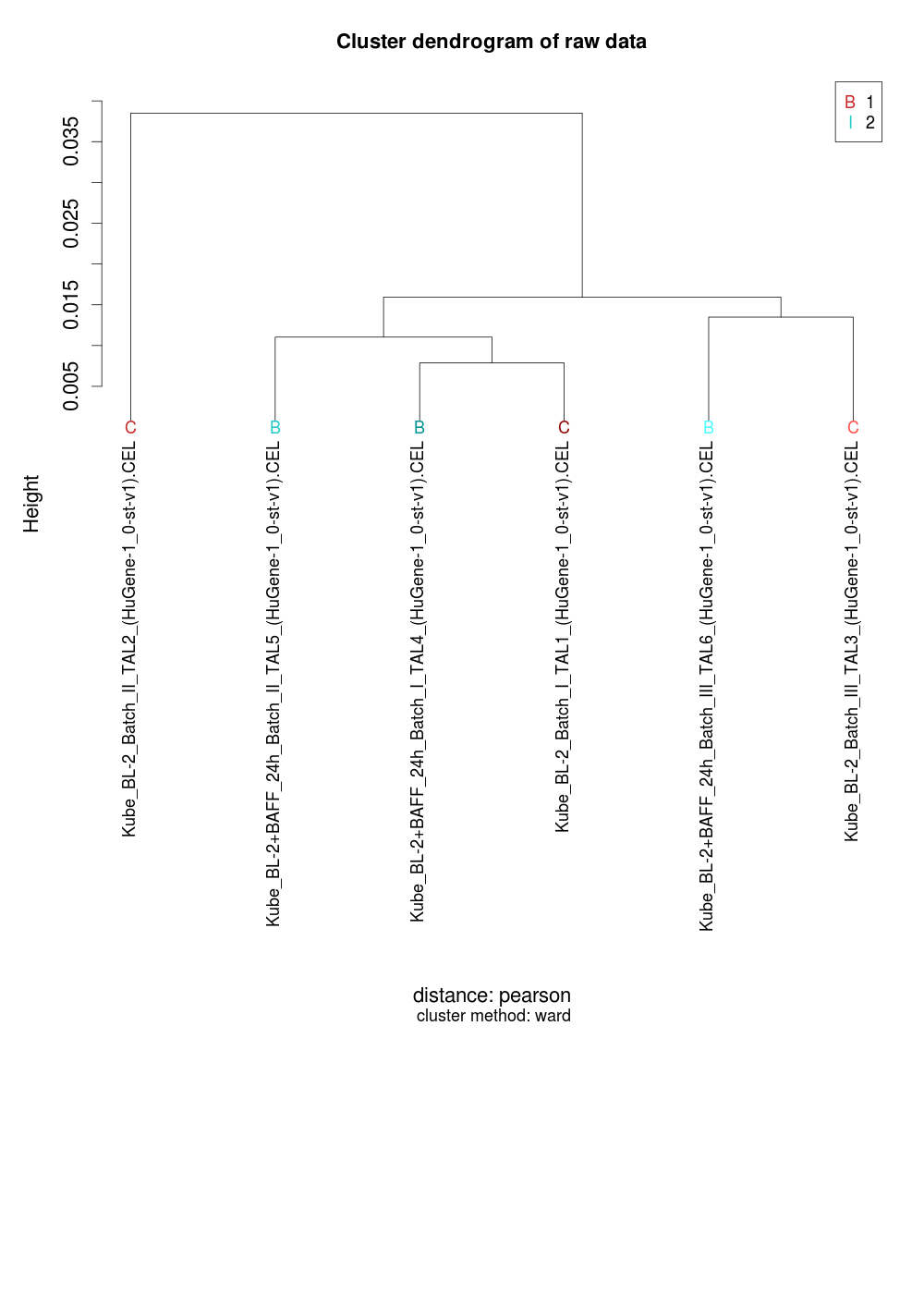

Supplement: S1 File — (GZ) [file pone.0197162.s001.tar.gz › RawDataCluster_pearson.png]

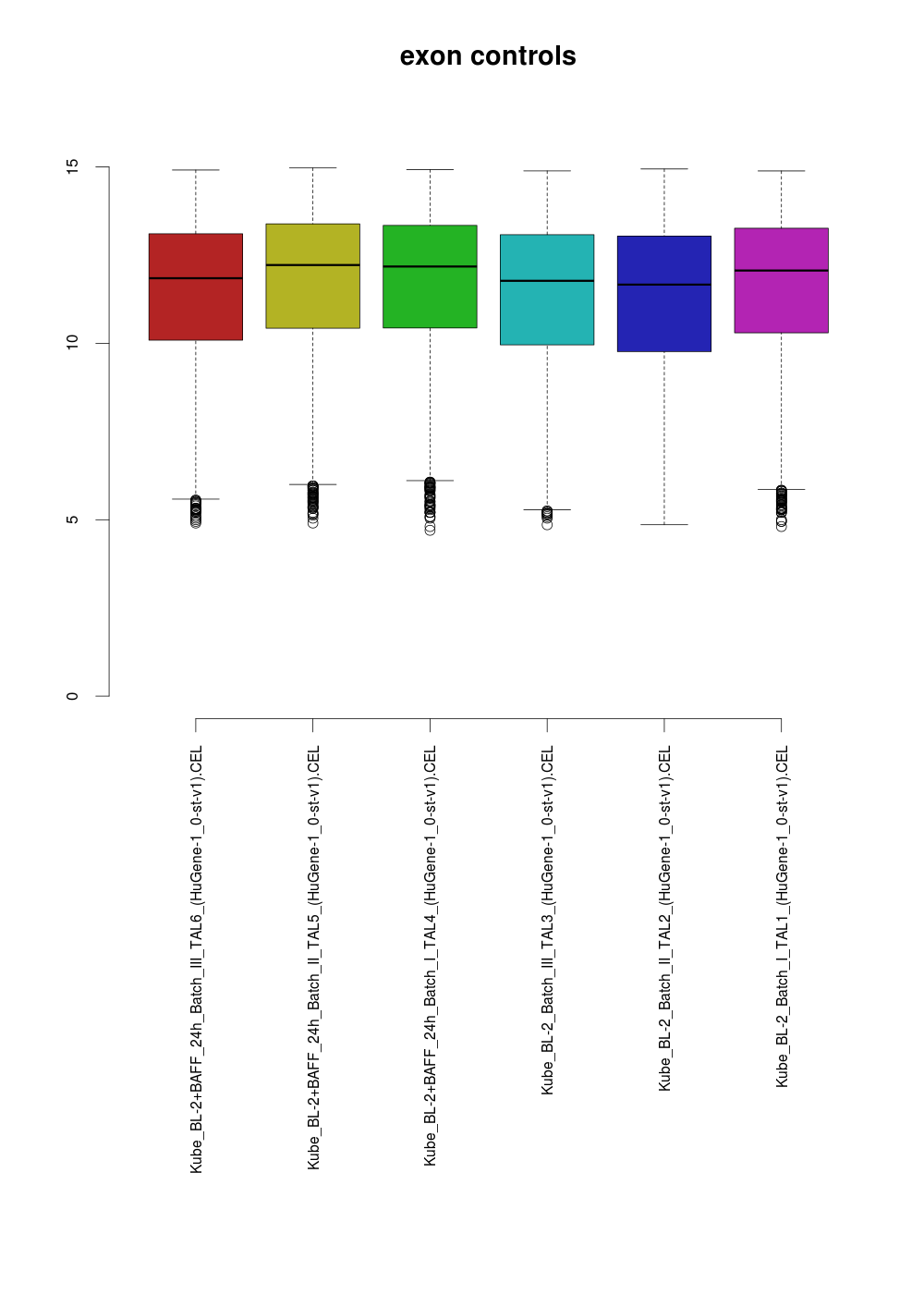

Supplement: S1 File — (GZ) [file pone.0197162.s001.tar.gz › RawDataEXONControlsBoxplot.png]

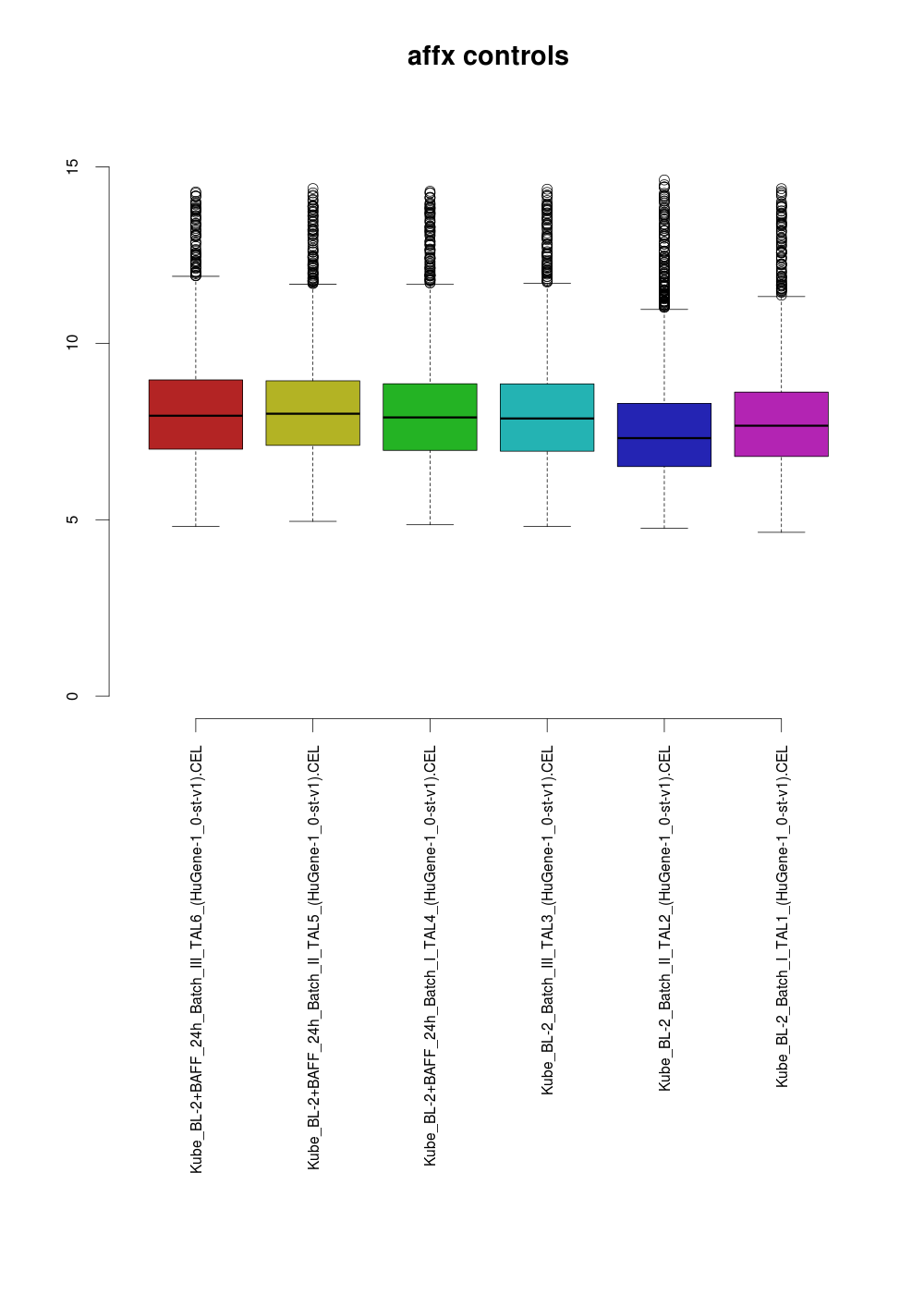

Supplement: S1 File — (GZ) [file pone.0197162.s001.tar.gz › RawDataAFFXControlsBoxplot.png]

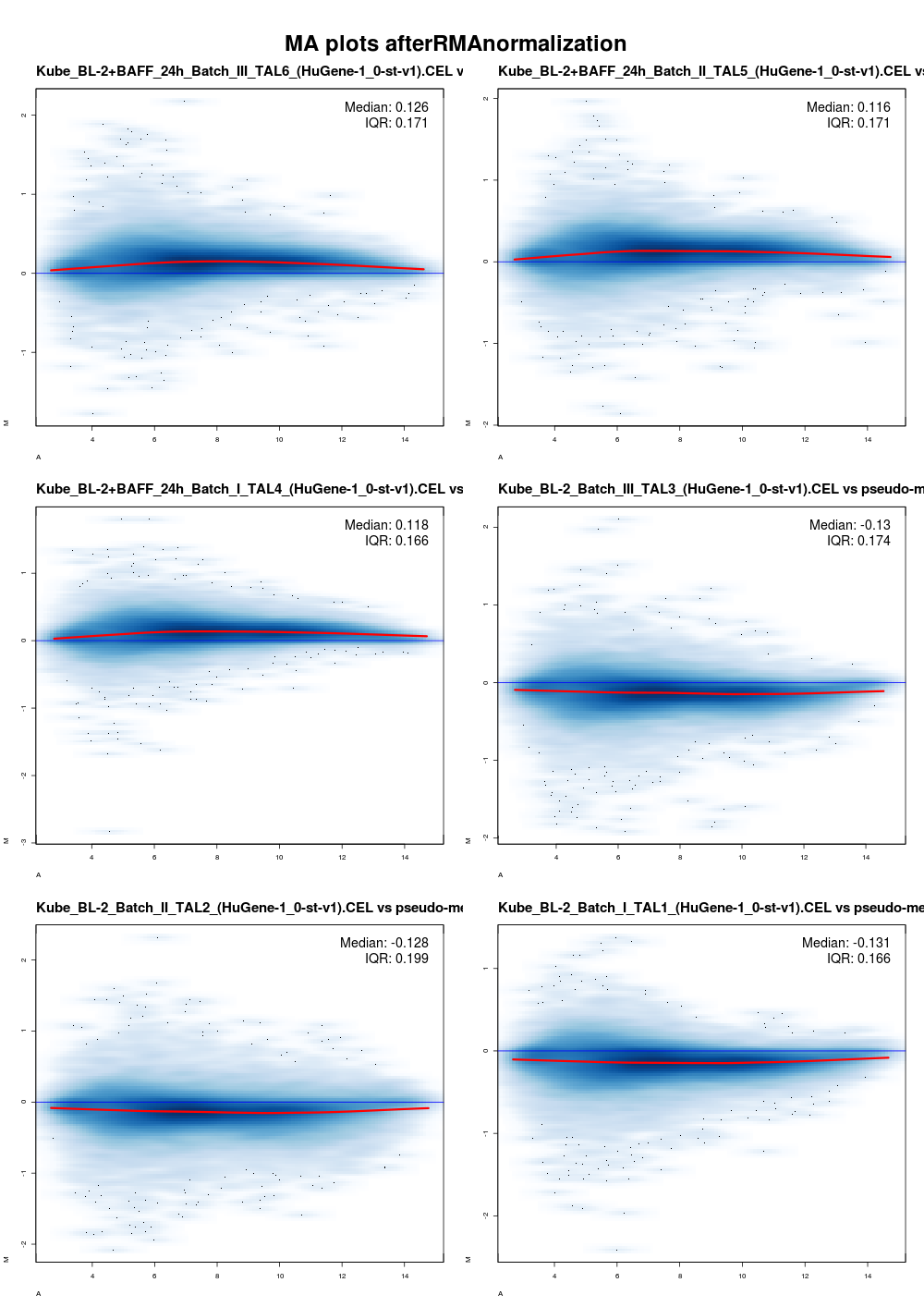

Supplement: S1 File — (GZ) [file pone.0197162.s001.tar.gz › NormDataMAplot.png]

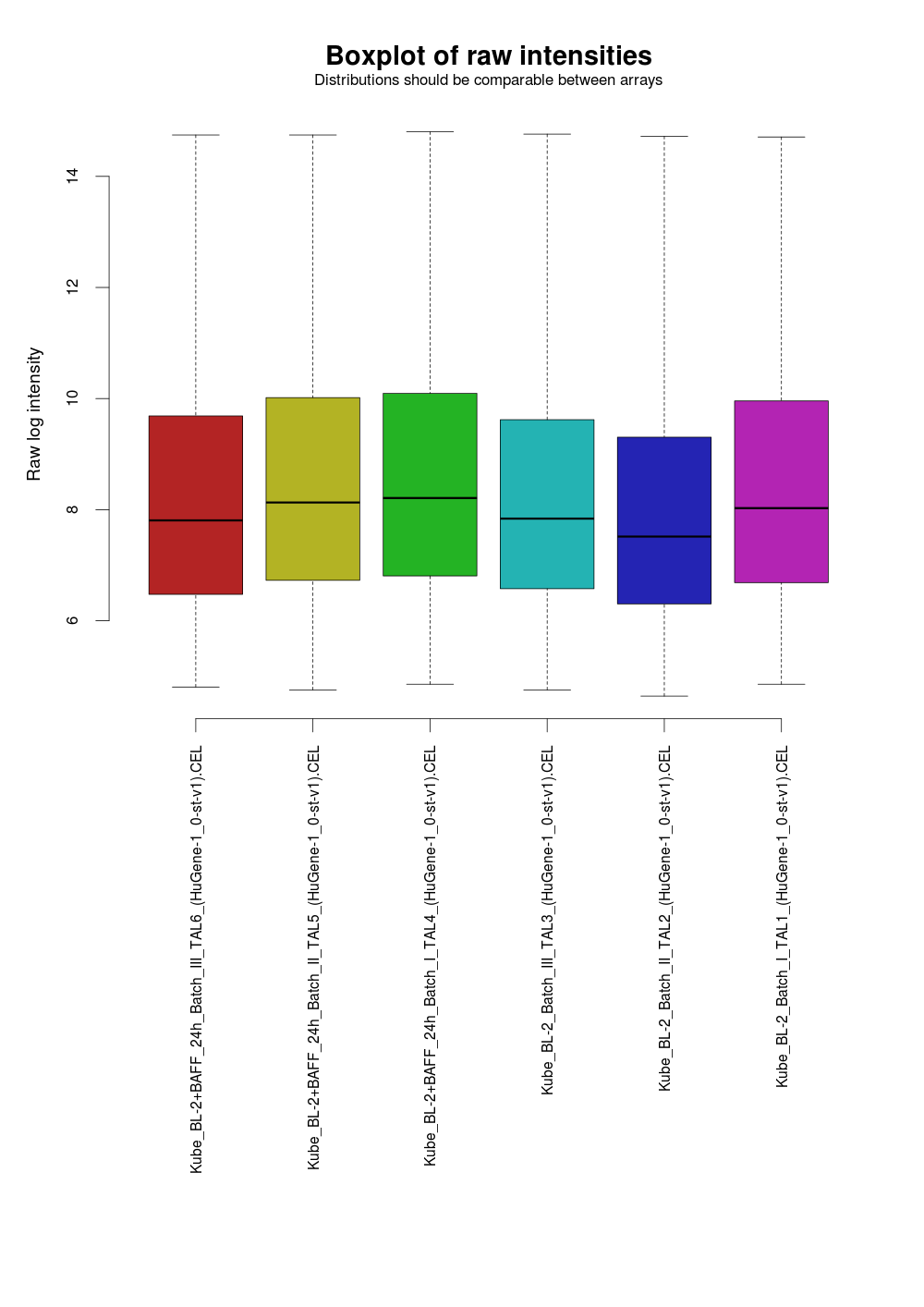

Supplement: S1 File — (GZ) [file pone.0197162.s001.tar.gz › RawDataBoxplot.png]

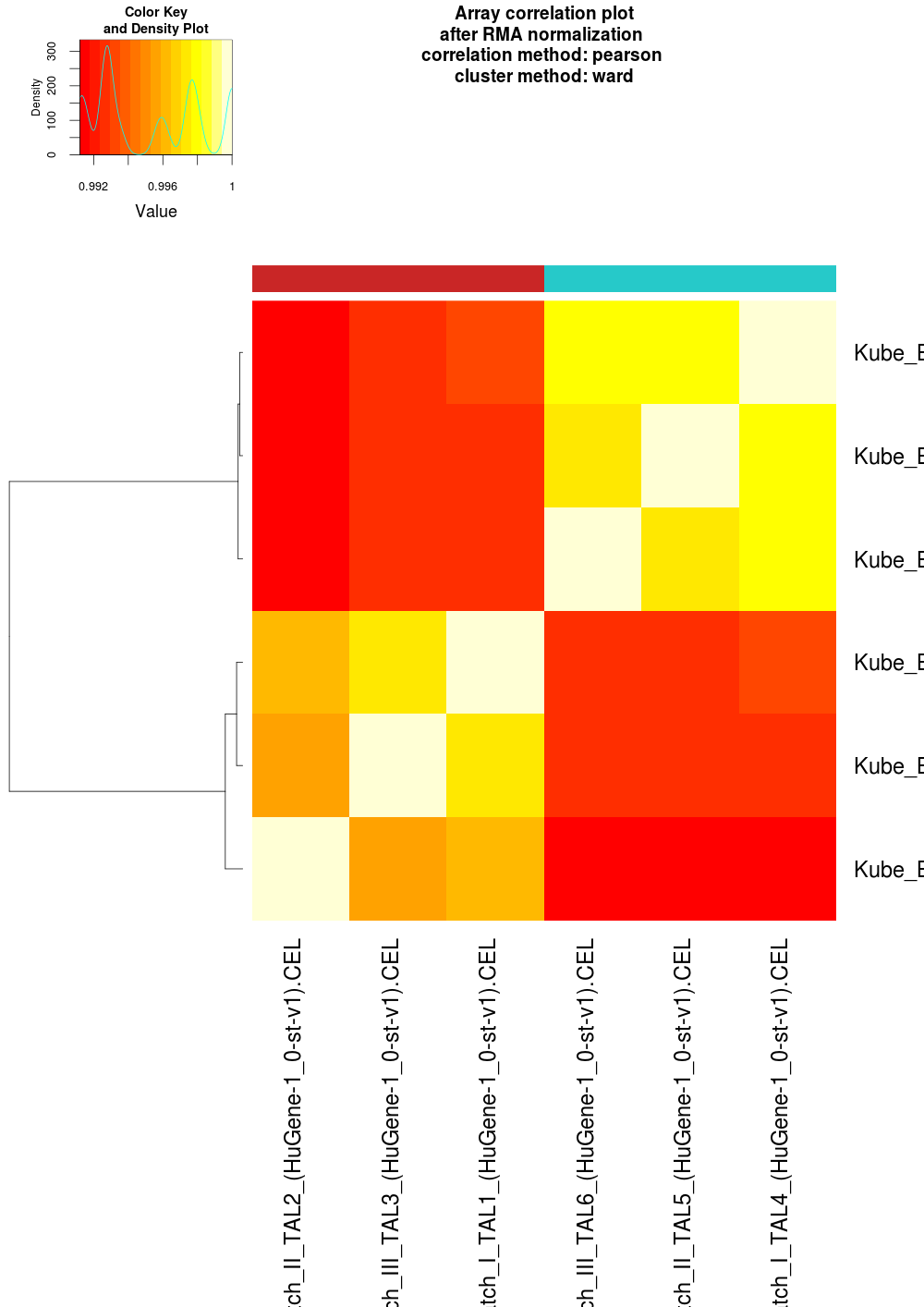

Supplement: S1 File — (GZ) [file pone.0197162.s001.tar.gz › NormDataArrayCorrelation.png]

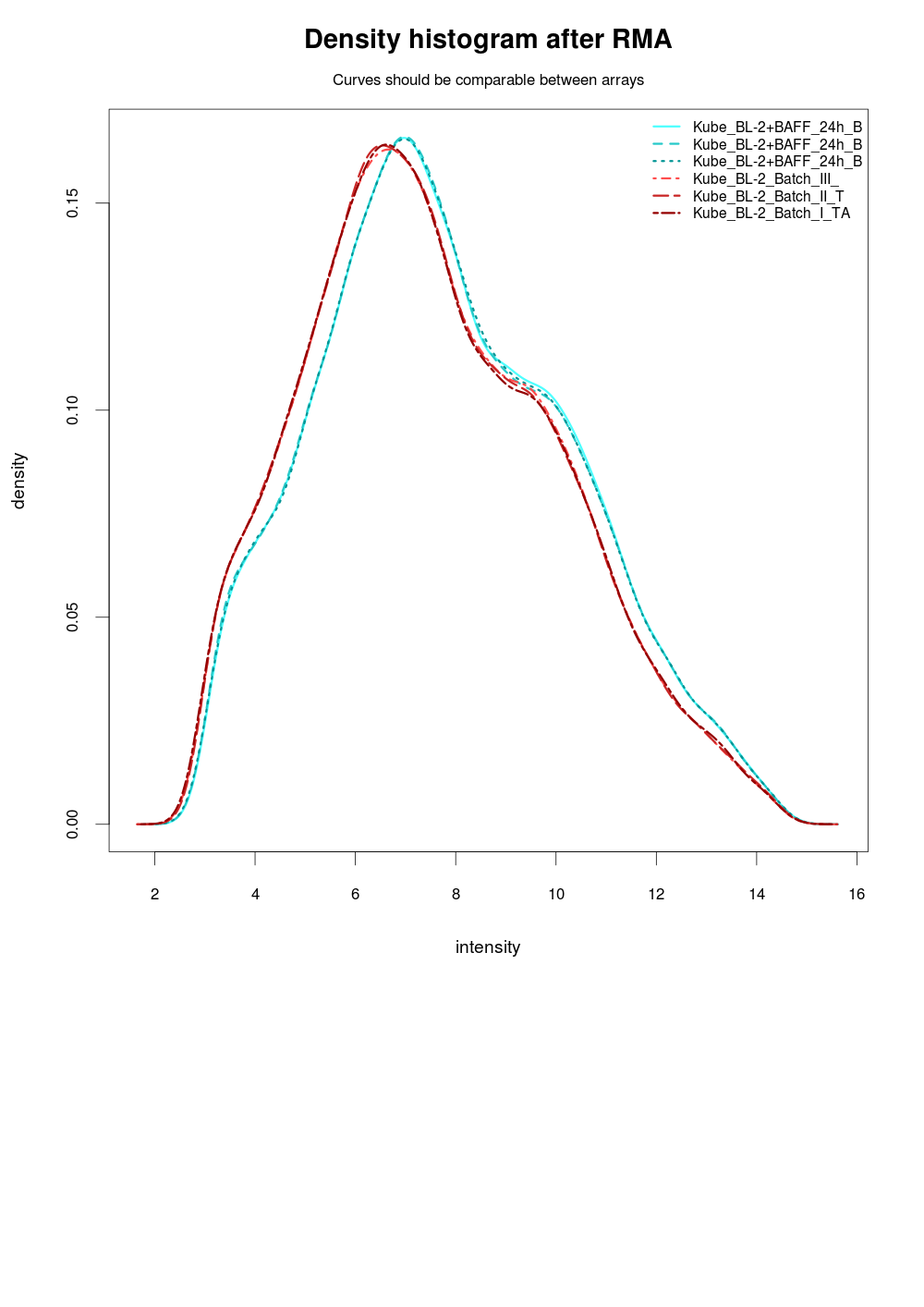

Supplement: S1 File — (GZ) [file pone.0197162.s001.tar.gz › NormDensityHistogram.png]

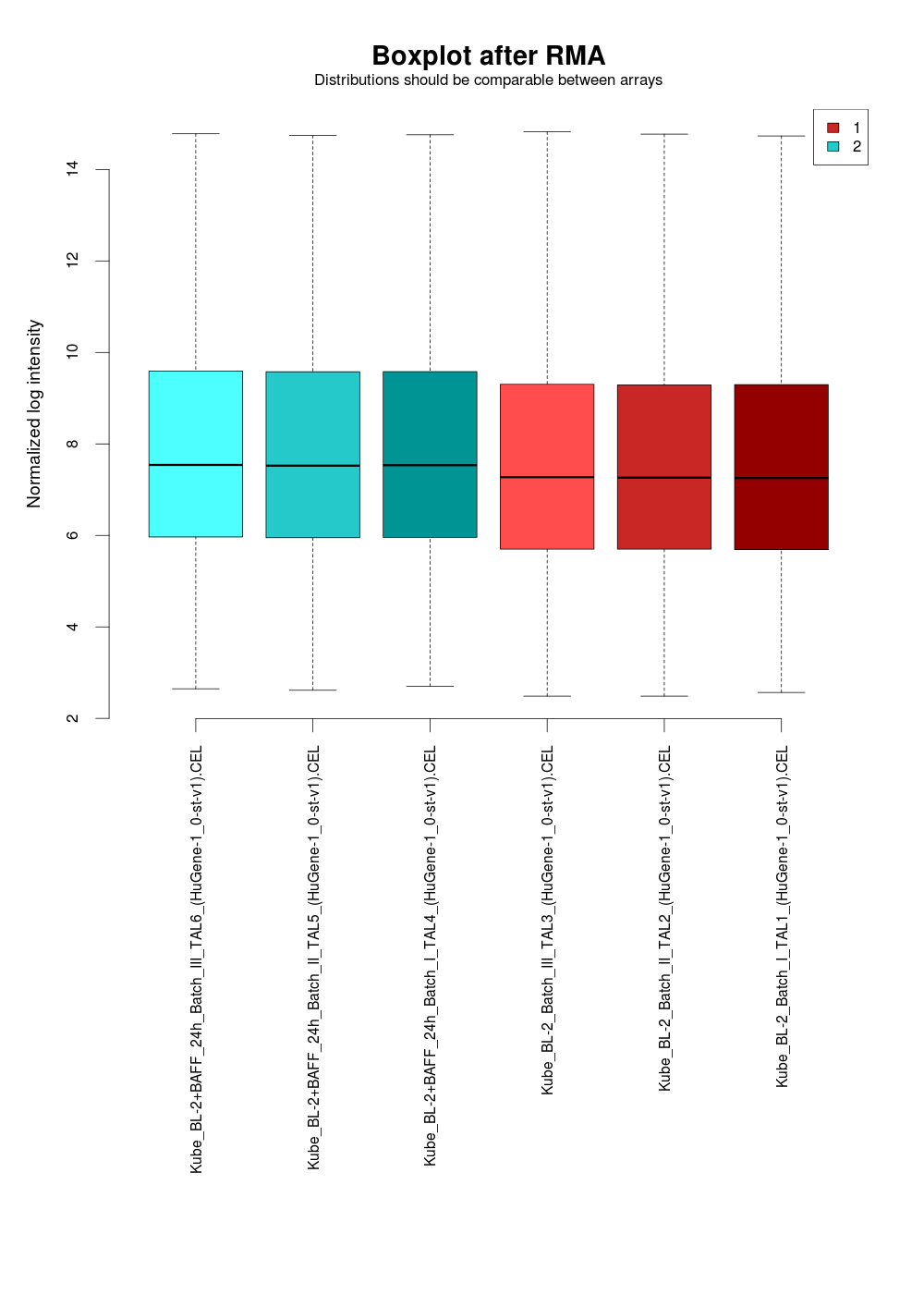

Supplement: S1 File — (GZ) [file pone.0197162.s001.tar.gz › NormDataBoxplot.png]

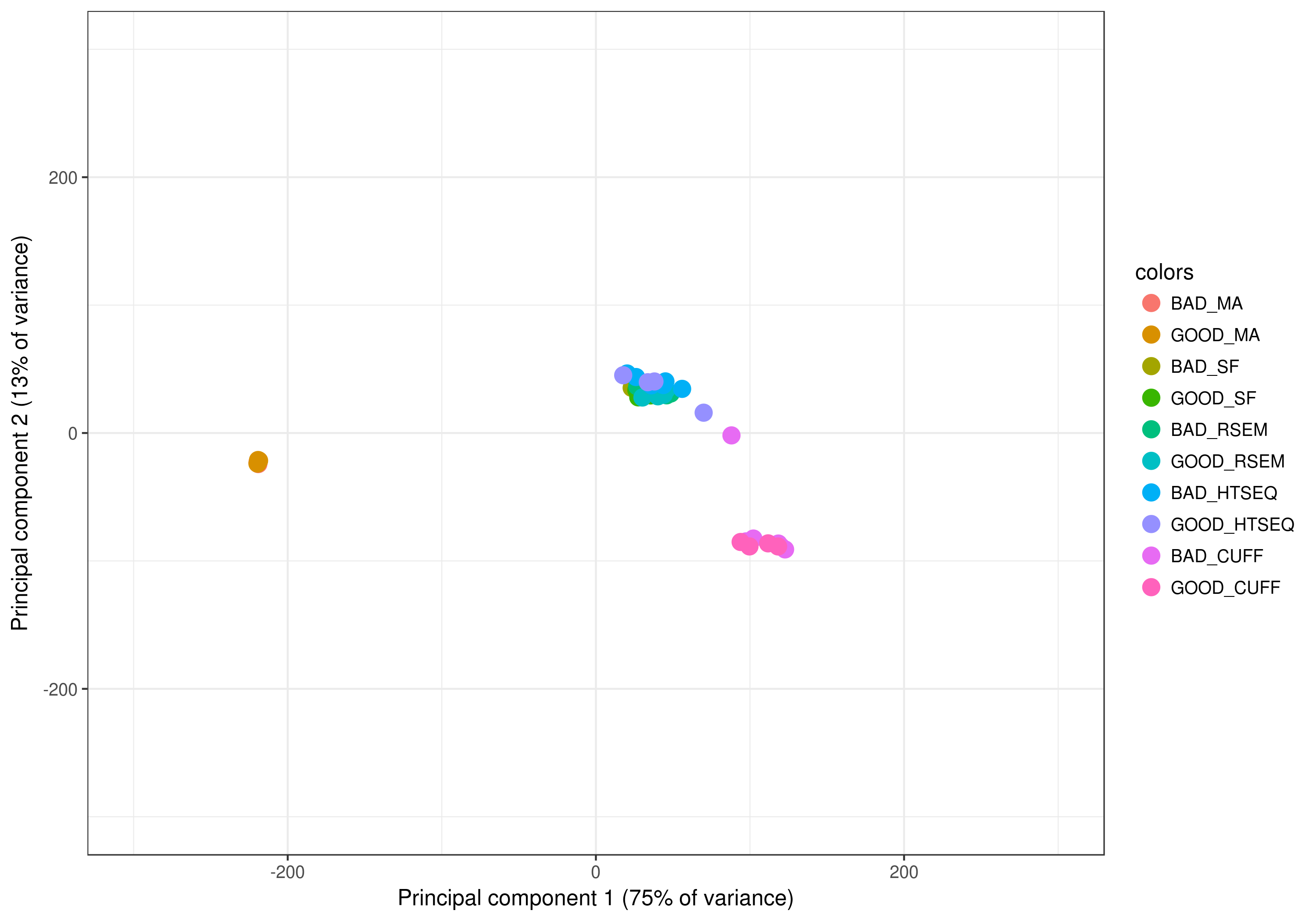

Supplement: S1 File — (GZ) [file pone.0197162.s001.tar.gz › S3b_Fig.png]

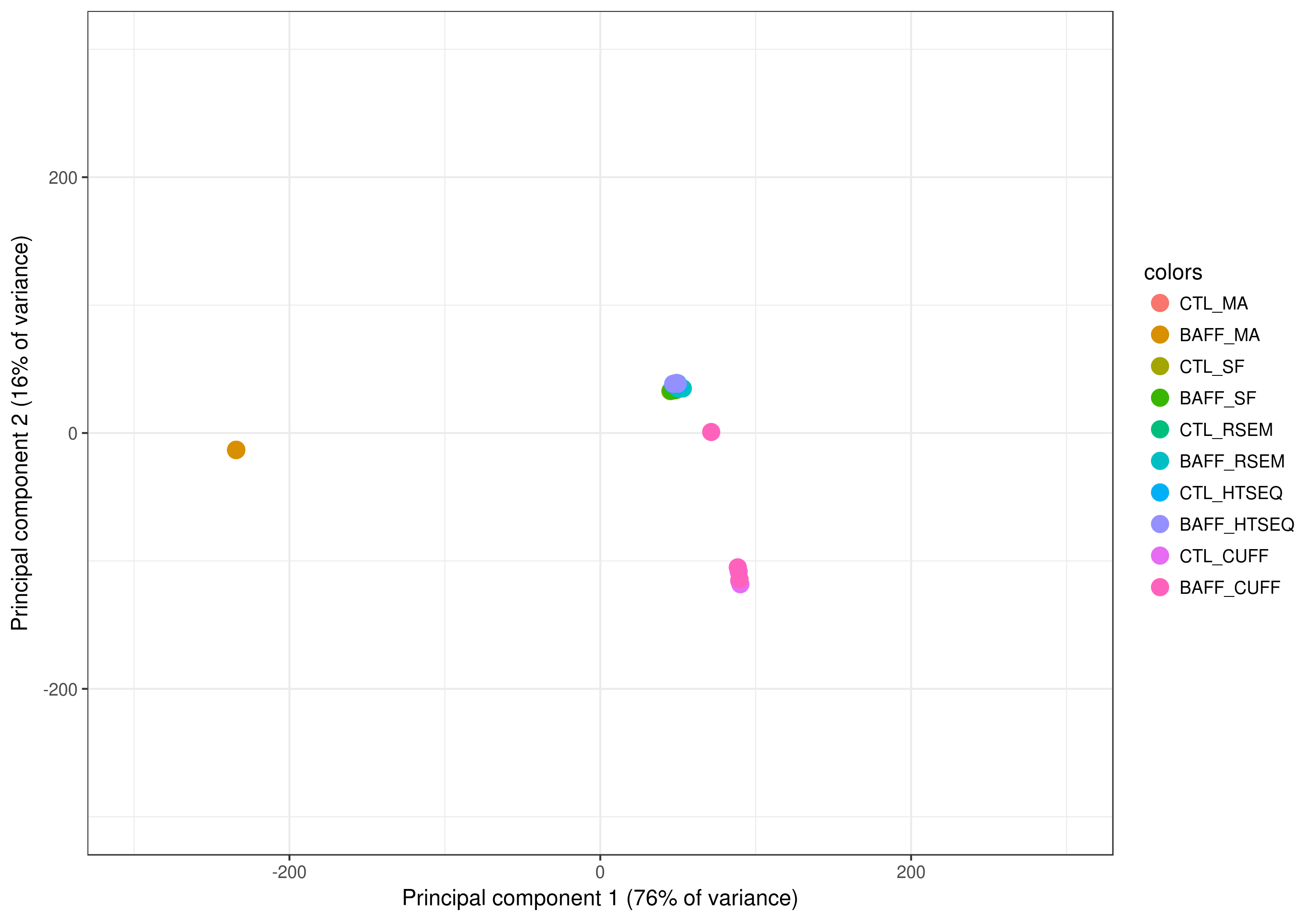

Supplement: S1 File — (GZ) [file pone.0197162.s001.tar.gz › S3a_Fig.png]

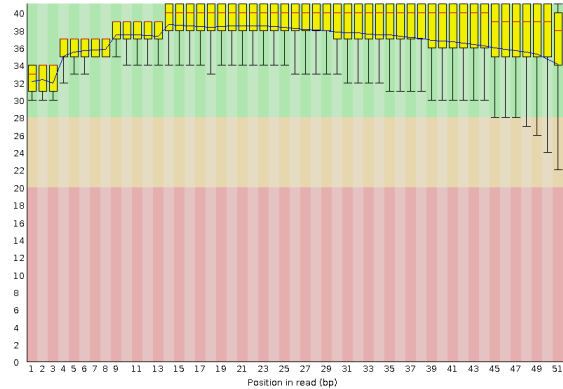

Supplement: S1 Fig — (PDF) [file pone.0197162.s006.pdf]

A

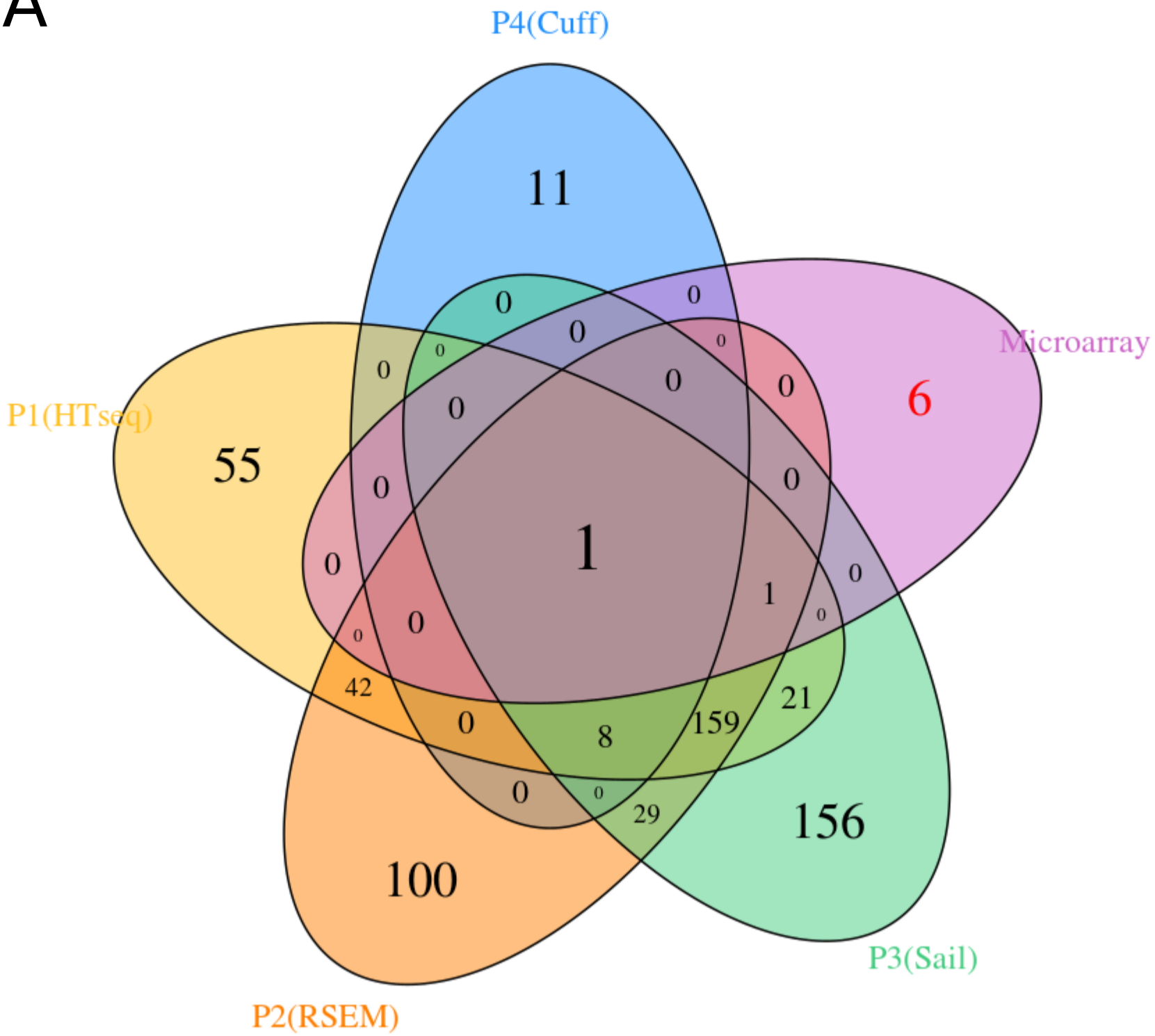

B

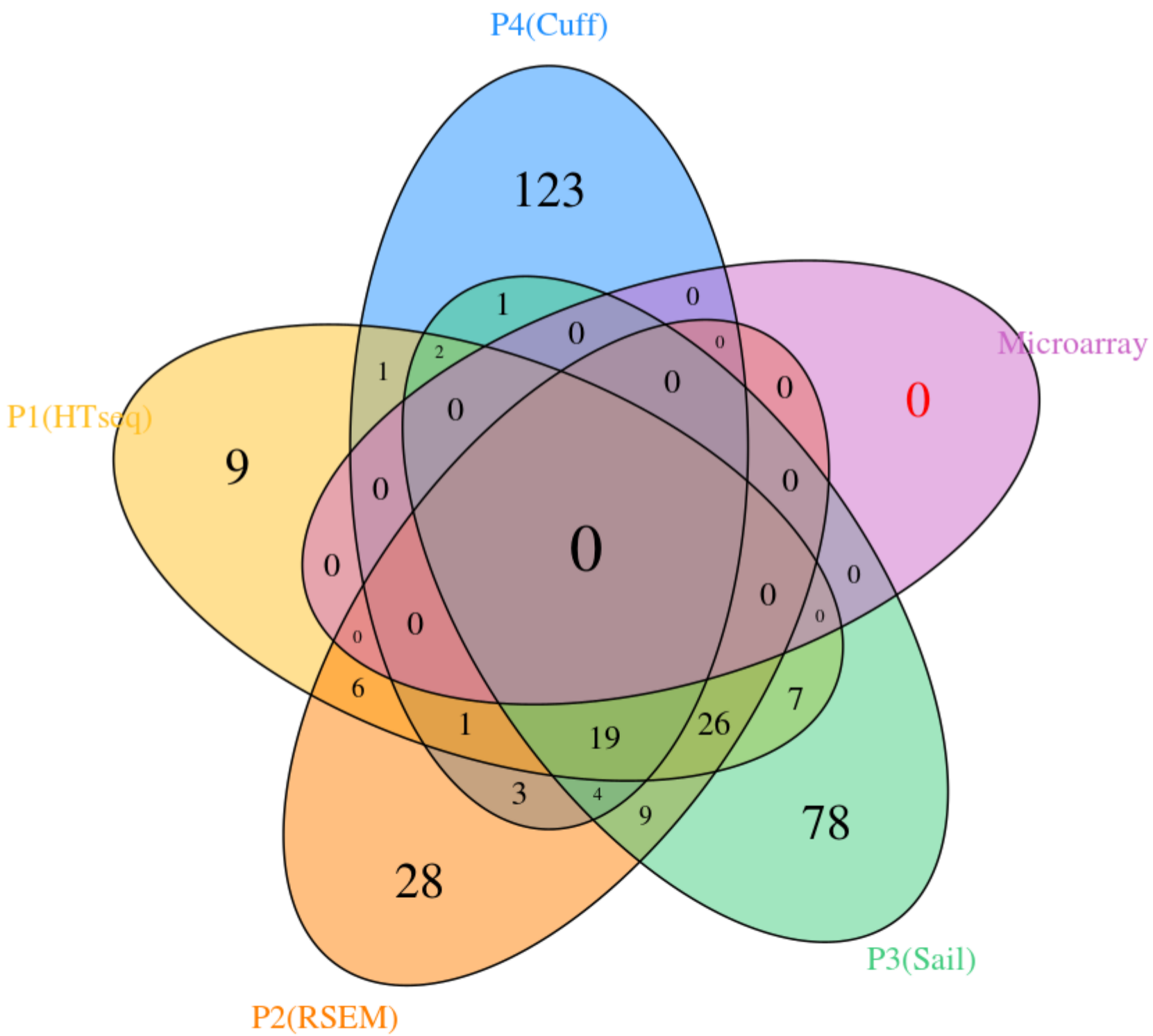

Supplement: S2 Fig — (PDF) [file pone.0197162.s007.pdf]
