# Supplementary material for: A comparative study of RNA-Seq and microarray data analysis on the two examples of rectal-cancer patients and Burkitt Lymphoma cells
Source: PLoS One. 2018 May 16;13(5):e0197162. doi: 10.1371/journal.pone.0197162 (PMC5955523; doi:10.1371/journal.pone.0197162)
Supplement: S1 Appendix — (DOC) [file pone.0197162.s002.doc]

RC

STAR+HTSEQ

[1] "Unqiue: 83.13+- 1.85"

[1] "Multi Mapped: 15.36+- 1.69"

[1] "Total Mapped: 98.49+- 0.35"

[1] "Unmapped: 1.51+- 0.35"

STAR+RSEM

[1] "Unqiue: 83.13+- 1.85"

[1] "Multi Mapped: 15.36+- 1.69"

[1] "Total Mapped: 98.49+- 0.35"

[1] "Unmapped: 1.51+- 0.35"

SAILFISH (transcriptome mapping of total RNA data)

[1] "Unqiue: 54.44+- 3.71"

[1] "Multi Mapped: 0+- 0"

[1] "Total Mapped: 54.44+- 3.71"

[1] "Unmapped: 45.56+- 3.71"

TOPHAT CUFFLINKs

[1] "Unqiue: 85.4+- 1.62"

[1] "Multi Mapped: 11.32+- 1.46"

[1] "Total Mapped: 96.73+- 0.4"

[1] "Unmapped: 3.27+- 0.4"

BL2

STAR+HTSEQ

[1] "Unqiue: 78.61+- 0.55"

[1] "Multi Mapped: 20.37+- 0.52"

[1] "Total Mapped: 98.98+- 0.05"

[1] "Unmapped: 1.02+- 0.05"

STAR_RSEM

[1] "Unqiue: 78.61+- 0.55"

[1] "Multi Mapped: 20.37+- 0.52"

[1] "Total Mapped: 98.98+- 0.05"

[1] "Unmapped: 1.02+- 0.05"

SAILFISH

[1] "Unqiue: 84.81+- 0.85"

[1] "Multi Mapped: 0+- 0"

[1] "Total Mapped: 84.81+- 0.85"

[1] "Unmapped: 15.19+- 0.85"

TOPHAT+cufllinks

[1] "Unqiue: 84.25+- 0.54"

[1] "Multi Mapped: 12.78+- 0.48"

[1] "Total Mapped: 97.02+- 0.1"

[1] "Unmapped: 2.98+- 0.1"

**S1 Appendix** : Mean readmapping-rates and their standard-deviation
