# Supplementary material for: A comparative study of RNA-Seq and microarray data analysis on the two examples of rectal-cancer patients and Burkitt Lymphoma cells
Source: PLoS One. 2018 May 16;13(5):e0197162. doi: 10.1371/journal.pone.0197162 (PMC5955523; doi:10.1371/journal.pone.0197162)
Supplement: S2 Appendix — (DOC) [file pone.0197162.s003.doc]

###########################################

# function to convert counts/fpkms/tpms

###########################################

countToTpm <- function(counts, effLen)

{

rate <- log(counts) - log(effLen)

denom <- log(sum(exp(rate)))

exp(rate - denom + log(1e6))

}

countToFpkm <- function(counts, effLen)

{

N <- sum(counts)

exp( log(counts) + log(1e9) - log(effLen) - log(N) )

}

fpkmToTpm <- function(fpkm)

{

exp(log(fpkm) - log(sum(fpkm)) + log(1e6))

}

countToEffCounts <- function(counts, len, effLen)

{

counts * (len / effLen)

}

**S2 Appendix** : R-functions for converting count and fpkm values into TPM
