# Supplementary material for: A comparative study of RNA-Seq and microarray data analysis on the two examples of rectal-cancer patients and Burkitt Lymphoma cells
Source: PLoS One. 2018 May 16;13(5):e0197162. doi: 10.1371/journal.pone.0197162 (PMC5955523; doi:10.1371/journal.pone.0197162)
Supplement: S1 Table — (DOC) [file pone.0197162.s004.doc]

| Sample | Number of single-end reads |
| --- | --- |
| P0022 | 50805747 |
| P0028 | 29184171 |
| P0048 | 46595774 |
| P0112 | 50688745 |
| P0123 | 47239853 |
| P0160 | 55961193 |
| P0291 | 39057478 |
| P0294 | 55749652 |
| P0300 | 43097237 |
| P0311 | 44128265 |
| Bl2Baff-1 | 15732861 |
| Bl2Baff-2 | 22320571 |
| Bl2Baff-3 | 22458073 |
| BL2-1 | 15560436 |
| BL2-2 | 16877337 |
| BL2-3 | 20680686 |

**S1 Table** Summary of RNA-Seq data
