# Supplementary material for: A comparative study of RNA-Seq and microarray data analysis on the two examples of rectal-cancer patients and Burkitt Lymphoma cells
Source: PLoS One. 2018 May 16;13(5):e0197162. doi: 10.1371/journal.pone.0197162 (PMC5955523; doi:10.1371/journal.pone.0197162)
Supplement: S2 Table — (DOC) [file pone.0197162.s005.doc]

**S2 Table**: Overview of Genes shared in common between our results and DEG results reported by Schrader et al.
