# Supplementary material for: A comparative study of RNA-Seq and microarray data analysis on the two examples of rectal-cancer patients and Burkitt Lymphoma cells
Source: PLoS One. 2018 May 16;13(5):e0197162. doi: 10.1371/journal.pone.0197162 (PMC5955523; doi:10.1371/journal.pone.0197162)
Supplement: S3 Fig — (PDF) [file pone.0197162.s008.pdf]

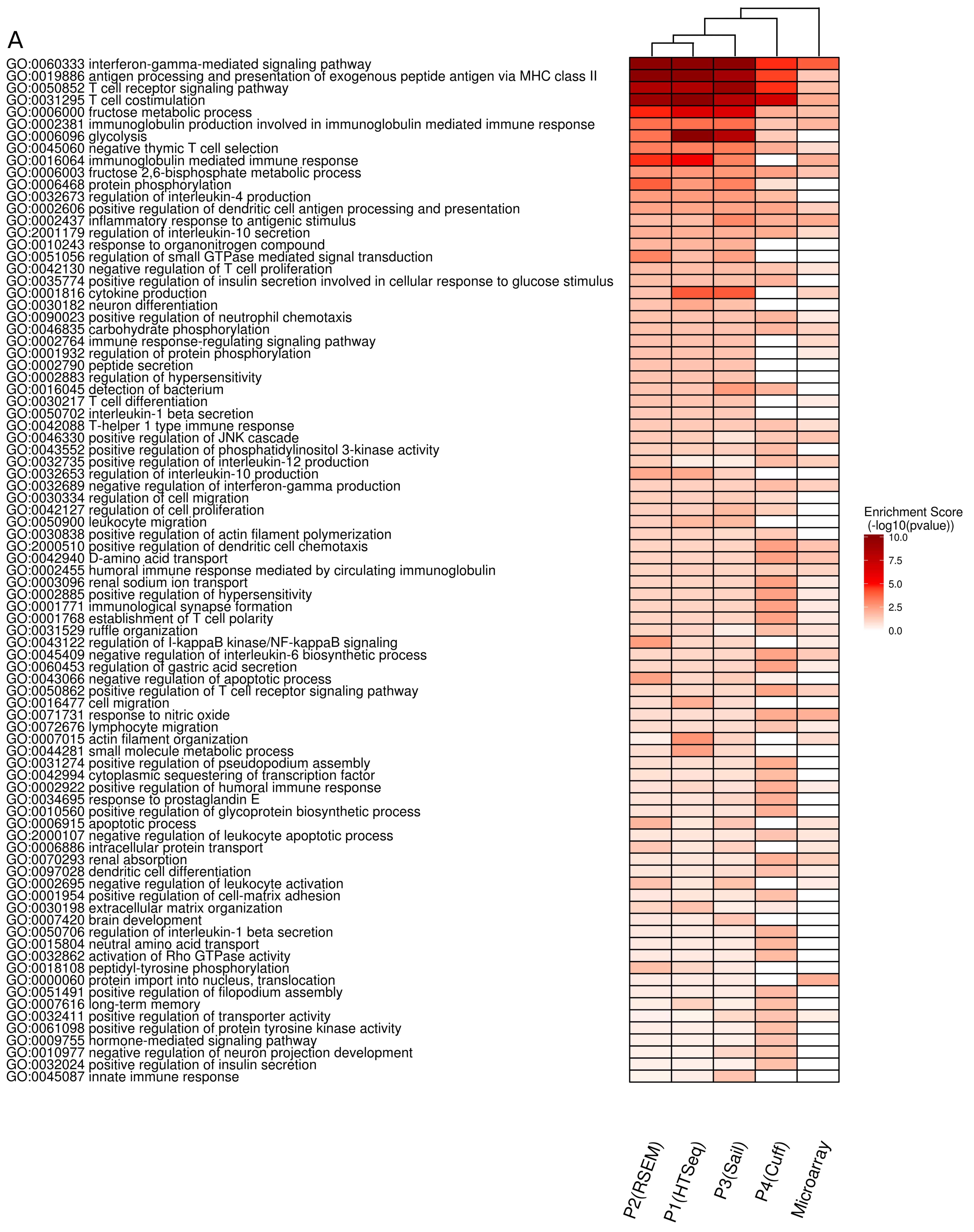
A
GO:0060333 interferon-gamma-mediated signaling pathway
GO:0019886 antigen processing and presentation of exogenous peptide antigen via MHC class II
GO:0050852 T cell receptor signaling pathway
GO:0031295 T cell costimulation
GO:0006000 fructose metabolic process
GO:0002381 immunoglobulin production involved in immunoglobulin mediated immune response
GO:0006096 glycolysis
GO:0045060 negative thymic T cell selection
GO:0016064 immunoglobulin mediated immune response
GO:0006003 fructose 2,6-bisphosphate metabolic process
GO:0006468 protein phosphorylation
GO:0032673 regulation of interleukin-4 production
GO:0002606 positive regulation of dendritic cell antigen processing and presentation
GO:0002437 inflammatory response to antigenic stimulus
GO:2001179 regulation of interleukin-10 secretion
GO:0010243 response to organonitrogen compound
GO:0051056 regulation of small GTPase mediated signal transduction
GO:0042130 negative regulation of T cell proliferation
GO:0035774 positive regulation of insulin secretion involved in cellular response to glucose stimulus
GO:0001816 cytokine production
GO:0030182 neuron differentiation
GO:0090023 positive regulation of neutrophil chemotaxis
GO:0046835 carbohydrate phosphorylation
GO:0002764 immune response-regulating signaling pathway
GO:0001932 regulation of protein phosphorylation
GO:0002790 peptide secretion
GO:0002883 regulation of hypersensitivity
GO:0016045 detection of bacterium
GO:0030217 T cell differentiation
GO:0050702 interleukin-1 beta secretion
GO:0042088 T-helper 1 type immune response
GO:0046330 positive regulation of JNK cascade
GO:0043552 positive regulation of phosphatidylinositol 3-kinase activity
GO:0032735 positive regulation of interleukin-12 production
GO:0032653 regulation of interleukin-10 production
GO:0032689 negative regulation of interferon-gamma production
GO:0030334 regulation of cell migration
GO:0042127 regulation of cell proliferation
GO:0050900 leukocyte migration
GO:0030838 positive regulation of actin filament polymerization
GO:2000510 positive regulation of dendritic cell chemotaxis
GO:0042940 D-amino acid transport
GO:0002455 humoral immune response mediated by circulating immunoglobulin
GO:0003096 renal sodium ion transport
GO:0002885 positive regulation of hypersensitivity
GO:0001771 immunological synapse formation
GO:0001768 establishment of T cell polarity
GO:0031529 ruffle organization
GO:0043122 regulation of I-kappaB kinase/NF-kappaB signaling
GO:0045409 negative regulation of interleukin-6 biosynthetic process
GO:0060453 regulation of gastric acid secretion
GO:0043066 negative regulation of apoptotic process
GO:0050862 positive regulation of T cell receptor signaling pathway
GO:0016477 cell migration
GO:0071731 response to nitric oxide
GO:0072676 lymphocyte migration
GO:0007015 actin filament organization
GO:0044281 small molecule metabolic process
GO:0031274 positive regulation of pseudopodium assembly
GO:0042994 cytoplasmic sequestering of transcription factor
GO:0002922 positive regulation of humoral immune response
GO:0034695 response to prostaglandin E
GO:0010560 positive regulation of glycoprotein biosynthetic process
GO:0006915 apoptotic process
GO:2000107 negative regulation of leukocyte apoptotic process
GO:0006886 intracellular protein transport
GO:0070293 renal absorption
GO:0097028 dendritic cell differentiation
GO:0002695 negative regulation of leukocyte activation
GO:0001954 positive regulation of cell-matrix adhesion
GO:0030198 extracellular matrix organization
GO:0007420 brain development
GO:0050706 regulation of interleukin-1 beta secretion
GO:0015804 neutral amino acid transport
GO:0032862 activation of Rho GTPase activity
GO:0018108 peptidyl-tyrosine phosphorylation
GO:0000060 protein import into nucleus, translocation
GO:0051491 positive regulation of filopodium assembly
GO:0007616 long-term memory
GO:0032411 positive regulation of transporter activity
GO:0061098 positive regulation of protein tyrosine kinase activity
GO:0009755 hormone-mediated signaling pathway
GO:0010977 negative regulation of neuron projection development
GO:0032024 positive regulation of insulin secretion
GO:0045087 innate immune response
Enrichment Score
(-log10(pvalue))
10.0
7.5
5.0
2.5
0.0
P2(RSEM)
P1(HTSeq)
P3(Sail)
P4(Cuff)
Microarray

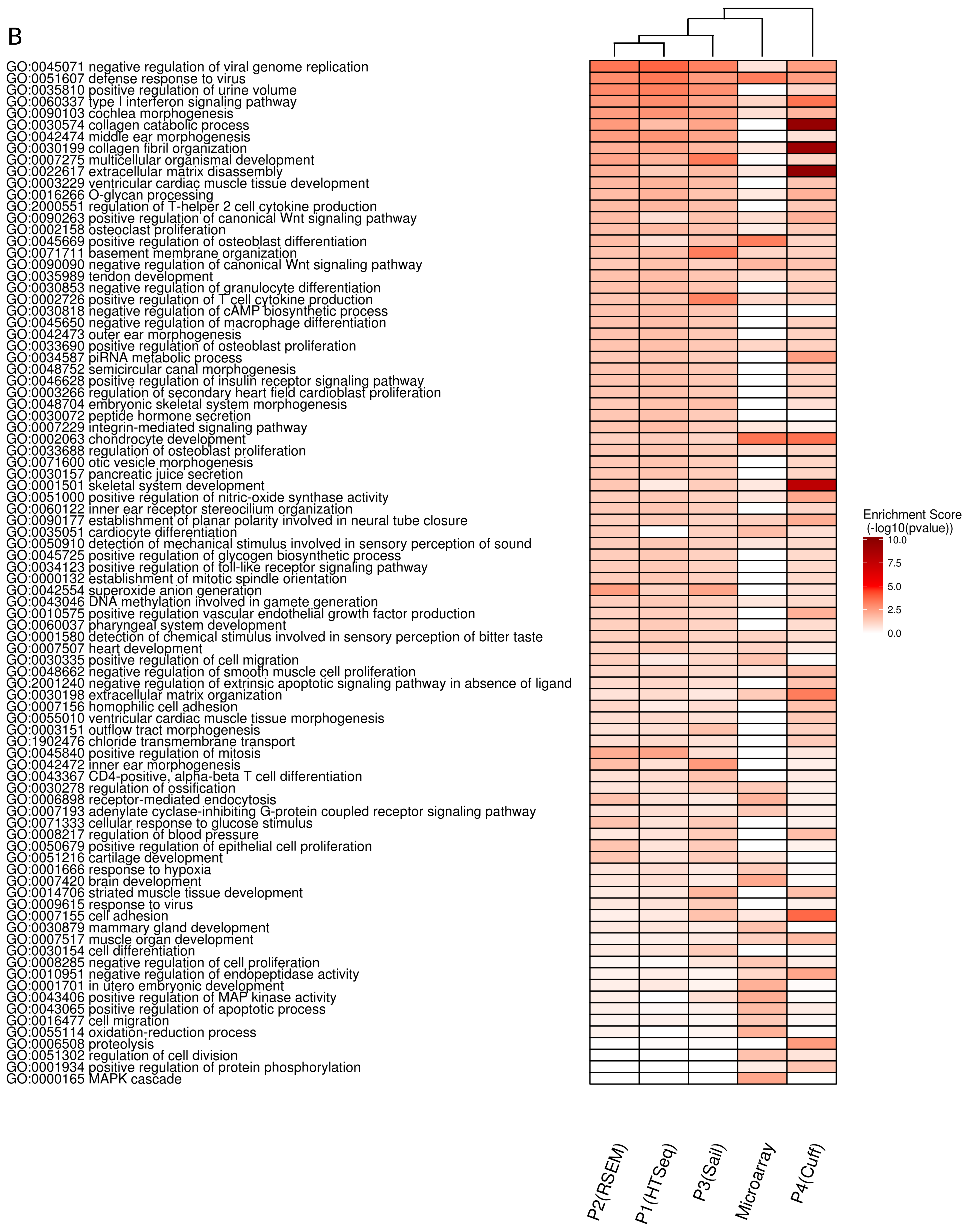
B
GO:0045071 negative regulation of viral genome replication
GO:0051607 defense response to virus
GO:0035810 positive regulation of urine volume
GO:0060337 type I interferon signaling pathway
GO:0090103 cochlea morphogenesis
GO:0030574 collagen catabolic process
GO:0042474 middle ear morphogenesis
GO:0030199 collagen fibril organization
GO:0007275 multicellular organismal development
GO:0022617 extracellular matrix disassembly
GO:0003229 ventricular cardiac muscle tissue development
GO:0016266 O-glycan processing
GO:2000551 regulation of T-helper 2 cell cytokine production
GO:0090263 positive regulation of canonical Wnt signaling pathway
GO:0002158 osteoclast proliferation
GO:0045669 positive regulation of osteoblast differentiation
GO:0071711 basement membrane organization
GO:0090090 negative regulation of canonical Wnt signaling pathway
GO:0035989 tendon development
GO:0030853 negative regulation of granulocyte differentiation
GO:0002726 positive regulation of T cell cytokine production
GO:0030818 negative regulation of cAMP biosynthetic process
GO:0045650 negative regulation of macrophage differentiation
GO:0042473 outer ear morphogenesis
GO:0033690 positive regulation of osteoblast proliferation
GO:0034587 piRNA metabolic process
GO:0048752 semicircular canal morphogenesis
GO:0046628 positive regulation of insulin receptor signaling pathway
GO:0003266 regulation of secondary heart field cardioblast proliferation
GO:0048704 embryonic skeletal system morphogenesis
GO:0030072 peptide hormone secretion
GO:0007229 integrin-mediated signaling pathway
GO:0002063 chondrocyte development
GO:0033688 regulation of osteoblast proliferation
GO:0071600 otic vesicle morphogenesis
GO:0030157 pancreatic juice secretion
GO:0001501 skeletal system development
GO:0051000 positive regulation of nitric-oxide synthase activity
GO:0060122 inner ear receptor stereocilium organization
GO:0090177 establishment of planar polarity involved in neural tube closure
GO:0035051 cardiocyte differentiation
GO:0050910 detection of mechanical stimulus involved in sensory perception of sound
GO:0045725 positive regulation of glycogen biosynthetic process
GO:0034123 positive regulation of toll-like receptor signaling pathway
GO:0000132 establishment of mitotic spindle orientation
GO:0042554 superoxide anion generation
GO:0043046 DNA methylation involved in gamete generation
GO:0010575 positive regulation vascular endothelial growth factor production
GO:0060037 pharyngeal system development
GO:0001580 detection of chemical stimulus involved in sensory perception of bitter taste
GO:0007507 heart development
GO:0030335 positive regulation of cell migration
GO:0048662 negative regulation of smooth muscle cell proliferation
GO:2001240 negative regulation of extrinsic apoptotic signaling pathway in absence of ligand
GO:0030198 extracellular matrix organization
GO:0007156 homophilic cell adhesion
GO:0055010 ventricular cardiac muscle tissue morphogenesis
GO:0003151 outflow tract morphogenesis
GO:1902476 chloride transmembrane transport
GO:0045840 positive regulation of mitosis
GO:0042472 inner ear morphogenesis
GO:0043367 CD4-positive, alpha-beta T cell differentiation
GO:0030278 regulation of ossification
GO:0006898 receptor-mediated endocytosis
GO:0007193 adenylate cyclase-inhibiting G-protein coupled receptor signaling pathway
GO:0071333 cellular response to glucose stimulus
GO:0008217 regulation of blood pressure
GO:0050679 positive regulation of epithelial cell proliferation
GO:0051216 cartilage development
GO:0001666 response to hypoxia
GO:0007420 brain development
GO:0014706 striated muscle tissue development
GO:0009615 response to virus
GO:0007155 cell adhesion
GO:0030879 mammary gland development
GO:0007517 muscle organ development
GO:0030154 cell differentiation
GO:0008285 negative regulation of cell proliferation
GO:0010951 negative regulation of endopeptidase activity
GO:0001701 in utero embryonic development
GO:0043406 positive regulation of MAP kinase activity
GO:0043065 positive regulation of apoptotic process
GO:0016477 cell migration
GO:0055114 oxidation-reduction process
GO:0006508 proteolysis
GO:0051302 regulation of cell division
GO:0001934 positive regulation of protein phosphorylation
GO:0000165 MAPK cascade
Enrichment Score
(-log10(pvalue))
10.0
7.5
5.0
2.5
0.0
P2(RSEM)
P1(HTSeq)
P3(Sail)
Microarray
P4(Cuff)
